# Plasmonic silver nanoshells for drug and metabolite detection

**DOI:** 10.1038/s41467-017-00220-4

**Published:** 2017-08-09

**Authors:** Lin Huang, Jingjing Wan, Xiang Wei, Yu Liu, Jingyi Huang, Xuming Sun, Ru Zhang, Deepanjali D. Gurav, Vadanasundari Vedarethinam, Yan Li, Ruoping Chen, Kun Qian

**Affiliations:** 1 School of Biomedical Engineering, Children’s Hospital of Shanghai, and Med-X Research Institute, Shanghai Jiao Tong University, Shanghai, 200030 People’s Republic of China; 2 Department of Chemistry, Shanghai University, Shanghai, 200444 People’s Republic of China; 3 Institute of Biophysics Key Laboratory of Interdisciplinary Research, Chinese Academy of Sciences, Beijing, 100101 People’s Republic of China

## Abstract

In-vitro metabolite and drug detection rely on designed materials-based analytical platforms, which are universally used in biomedical research and clinical practice. However, metabolic analysis in bio-samples needs tedious sample preparation, due to the sample complexity and low molecular abundance. A further challenge is to construct diagnostic tools. Herein, we developed a platform using silver nanoshells. We synthesized SiO_2_@Ag with tunable shell structures by multi-cycled silver mirror reactions. Optimized nanoshells achieved direct laser desorption/ionization mass spectrometry in 0.5 μL of bio-fluids. We applied these nanoshells for disease diagnosis and therapeutic evaluation. We identified patients with postoperative brain infection through daily monitoring and glucose quantitation in cerebrospinal fluid. We measured drug distribution in blood and cerebrospinal fluid systems and validated the function of blood-brain/cerebrospinal fluid-barriers for pharmacokinetics. Our work sheds light on the design of materials for advanced metabolic analysis and precision diagnostics.

## Abstract (web summary)

Preparation of samples for diagnosis can affect the detection of biomarkers and metabolites. Here, the authors use a silver nanoparticle plasmonics approach for the detection of biomarkers in patients as well as investigate the distribution of drugs in serum and cerebral spinal fluid.

## Introduction

In-vitro metabolic diagnosis relies on designed materials-based analytical platforms for detection of selected metabolites in biological samples, which has a key role for disease detection and therapeutic evaluation in clinics^1, 2^. Currently, both inorganic and organic materials have been used for metabolic analysis by selected spectroscopy methods, including nuclear magnetic resonance spectroscopy, biochemical analyzers, and mass spectrometry (MS) et al.^1–5^. In contrast with other methods, MS enjoys unique advantages of high accuracy, sensitivity, resolution, and throughput. However, the efficacy of MS and other spectroscopy methods is determined by rigorous sample pre-treatment procedures for enrichment or purification^2, 6–9^, due to the high sample complexity and low molecular abundance in biological samples. To date, MS-based analysis of metabolites in bio-fluids is still unsatisfactory in terms of selectivity, speed, costs, and reproducibility for clinical use. Therefore, to address the challenges in the sample pre-treatment, a designer material platform is in urgent demand for advanced in-vitro metabolic diagnosis.

Matrix-assisted laser desorption/ionization (MALDI) MS has displayed great superiority considering its fast analysis of analytes in seconds, accurate mass measurement for identification, simple sample preparation for broad application, and high sensitivity with low costs for practical use^1, 2, 5^. Inorganic particles (e.g., silicon^10, 11^, carbon^12, 13^, metal^14–16^, and metal oxide^17, 18^) have been preferred matrices in LDI MS for small metabolites as reported by research groups globally including ours. These particles can control the analytical variations and background signals in the mass region below *m*/*z* of 500, achieving the qualitative analysis of small metabolites, such as carbohydrates and amino acids^6, 7, 19^. Further coupling LDI MS with isotopic quantification, designed particles enabled metabolic analysis of rare cells, e.g., circulating tumor cells^17^. Notably, the LDI performance of particles is far from ideal for selective detection of small metabolites, dealing with complex bio-fluids in real case. As a result, the application of particle-assisted LDI MS for clinical diagnostics is very challenging and needs rational design of materials for use.

Plasmonic particles consist of noble metals including but not limited to Au, Ag, Pt, and Pd, which provide unique surface plasmon resonance and hot carriers^20, 21^ under laser irradiation ideal for matrix use. Despite that there have been many reports on the application of plasmonic particles for LDI MS^6, 7, 14, 15, 19^, most of current work are focused on the use of solid plasmonic particles in diverse shapes and there are very few reports on plasmonic nanoshells. Plasmonic Au nanoshells with nanoscaled surface roughness and higher hot carriers production over solid Au particles^16, 22^ have been demonstrated to be better candidates for LDI MS detection of small metabolites, whereas their application is very limited in complex bio-fluids for diagnostic purpose^23^. Notably, the analytical performance of plasmonic nanoshells depends on the selection of noble metals to adsorb ultraviolet (UV) laser and optimized shell structure for LDI MS detection. Designer plasmonic nanoshells would achieve efficient LDI MS for metabolic analysis in real case for clinical applications but have not been developed so far.

In this work, we report a platform based on designer plasmonic silver nanoshells for direct detection of small metabolites in clinical in-vitro metabolic diagnostics. We first synthesized a series of SiO_2_@Ag core-shell particles with tunable nanoshell structures through multi-cycled silver mirror reactions on the surface of SiO_2_ nanoparticles. The optimized plasmonic silver nanoshells as new matrices allowed fast, multiplex, sensitive, and selective LDI MS detection of small metabolites in 0.5 μL of bio-fluids without enrichment or purification. Furthermore, coupling with isotopic quantification of selected metabolites, we demonstrated the use of these silver nanoshells for disease detection and therapeutic evaluation in clinics. For disease detection, we identified patients with postoperative brain infection through glucose quantitation and daily monitoring by cerebrospinal fluid (CSF) analysis. For therapeutic evaluation, we investigated drug distribution in blood and CSF systems and validated the function and permeability of blood–brain/CSF-barriers, during therapeutic treatment of patients with cerebral edema for pharmacokinetics study. Our work sheds light on the design of materials for high-performance metabolic analysis and precision diagnostics in real cases.

## Results

### Preparation and characterization of silver nanoshells

**Fig. 1:**
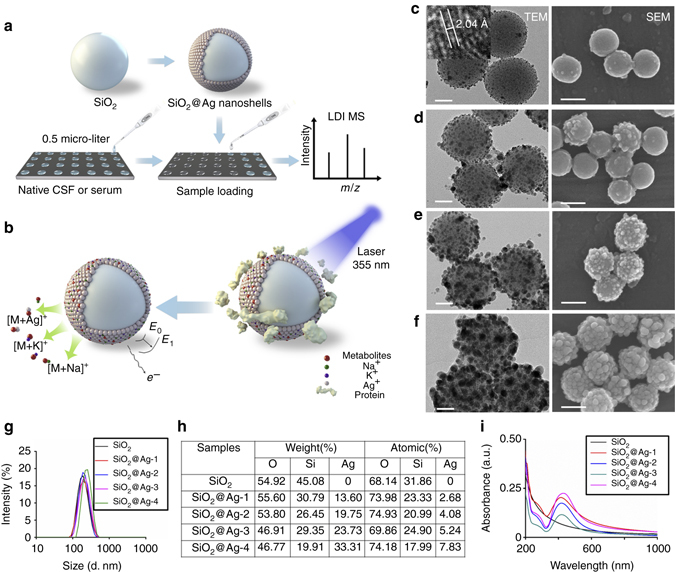
Schematic workflow and material characterization. Schematic diagrams of **a** experimental workflow and **b** LDI MS process using SiO_2_@Ag nanoshells as matrix. Electron micrograph images of **c** SiO_2_@Ag-1, **d** SiO_2_@Ag-2, **e** SiO_2_@Ag-3, and **f** SiO_2_@Ag-4, including TEM images of particles and HRTEM showing silver crystal lattice (*inset* of **c**), and SEM images of particles. **g** average size distributions of SiO_2_ and SiO_2_@Ag particles in water by DLS; **h** elementary composition analysis of SiO_2_ and SiO_2_@Ag particles; **i** UV-Vis absorption spectra of SiO_2_ and SiO_2_@Ag particles. *Scale bars*: 50 nm (TEM); 150 nm (SEM)

We synthesized silver nanoshells (also denoted SiO_2_@Ag) through a hard templating method (Fig. 1a), and the shell structures can be controlled using multi-cycled silver mirror reactions for LDI MS (Fig. 1b). The reaction temperature and time were critical and optimized in the synthesis process (Supplementary Fig. 1, see also “Methods” for details). We prepared mono-dispersed silica particles by the Stöber method with an average size of 181.8 ± 12.53 nm and polydispersity index (PDI) of 0.203 ± 0.112 as the core materials (Supplementary Table 1; Supplementary Fig. 2). After the first cycle of silver mirror reaction, we anchored the silver nanoparticles with a size of ~2–4 nm onto the surface of silica particles (SiO_2_@Ag-1, Fig. 1c) and high-resolution transmission electron microscopy (HRTEM) demonstrated the typical inter planar spacing of 2.04 Å for silver composites along [2, 0, 0] direction (*inset* of Fig. 1c)^24, 25^. After multiple cycles (two cycles for SiO_2_@Ag-2, three cycles for SiO_2_@Ag-3, and four cycles for SiO_2_@Ag-4) of silver mirror reactions, we observed the gradual increase of shell thickness and silver nanoparticle size for SiO_2_@Ag-2 (Fig. 1d), SiO_2_@Ag-3 (Fig. 1e), and SiO_2_@Ag-4 (Fig. 1f), due to the seed-mediated growth process^26, 27^. We characterized the morphologies of SiO_2_@Ag, showing controlled nanoscaled surface roughness and nano-crevices under scanning electron microscopy (SEM) by the multi-cycled silver mirror reactions (Fig. 1c–f). As the electricity/heat insulating materials, the silica cores of SiO_2_@Ag contributed to produce more hot carriers^20–22, 28^ and retained the heat during LDI process, whereas the silver shell can be excited under 355 nm of laser^22, 23, 28^. Moreover, as shown in the elemental mapping analysis (Supplementary Fig. 3)^29, 30^, the distribution of carbon (from the small metabolites), silver (from the shell, affording similar silver nanoparticles densities on surface to Fig. 1e with silver loading ratio of 22.80% by weight), and silicon (from the core) in the nanoshells–metabolites hybrids demonstrated the small molecules were trapped by nano-crevices on the surface of silver nanoshells, which would be crucial for efficient LDI of small metabolites.

We also examined the important structural parameters for LDI MS matrix use including the dispersity, zeta potential, surface area, composition, and light-absorbance of all prepared SiO_2_@Ag. In the dynamic light scattering (DLS) experiments, all SiO_2_@Ag showed fine dispersity in water with PDI of 0.105 ± 0.064–0.313 ± 0.044 (Supplementary Table 1), and the particle sizes increased from 185.8 ± 0.611 nm to 289.7 ± 74.19 nm (Fig. 1g) with multi-cycled silver mirror reactions, consistent with transmission electron microscopy (TEM) and SEM results confirming the controlled synthesis of SiO_2_@Ag. We obtained similar results for three batches of materials in parallel and demonstrated the reproducibility of the synthetic method with no significant difference (*p* < 0.05, Supplementary Fig. 4a). SiO_2_@Ag particles were negatively charged with zeta potentials between −37.4 ± 1.760 mV and −22.2 ± 1.130 mV (Supplementary Table 1; Supplementary Fig. 5), beneficial for the formation of ion layers on the surface towards Na^+^/K^+^/Ag^+^ adduction. In the nitrogen adsorption analysis, we obtained type IV isotherms (Supplementary Fig. 6)^31–34^, suggesting a stacking mesoporous structure on the material surface. We calculated surface area of SiO_2_@Ag based on the multipoint Brunauer, Emmett, and Teller (BET) model^31, 35^ and SiO_2_@Ag enjoyed larger surface area (20.72 ± 0.115–24.20 ± 0.069 m^2^ g^−1^) compared with the bare SiO_2_ particles (18.58 ± 0.095 m^2^ g^−1^) because of the increased surface roughness and introduction of silver (*p* < 0.05, Supplementary Table 1). The increasing average size of silver nanoshells did not correlate to a similar increase in surface area due to the change of density similar to previous reports^36, 37^. To investigate the composition of materials, we recorded corresponding energy dispersive X-Ray (EDX) spectra (Fig. 1h; Supplementary Fig. 7), yielding an increasing silver loading ratio (by weight) of 0% for SiO_2_, 13.6% for SiO_2_@Ag-1, 19.75% for SiO_2_@Ag-2, 23.73% for SiO_2_@Ag-3 (consistent with Supplementary Fig. 3e), and 33.31% for SiO_2_@Ag-4.

To explore the light absorbance of particles, we performed ultraviolet-visible (UV-Vis) spectroscopy analysis in Fig. 1i and Supplementary Fig. 4b. The pure silica particles did not have any UV-Vis absorption peaks similar to reference reports^23, 38^, whereas the SiO_2_@Ag-1/2/3 particles displayed the absorption peak at ~436 ± 2.45 nm due to the Mie plasmon resonance excitation from the silver nanoparticles^38, 39^. After the fourth reduction cycle, the absorption peak red shifted to ~443 ± 1.63 nm for SiO_2_@Ag-4 (*p* < 0.05), due to the enhanced plasmon coupling and aggregation of excess silver on the surface of silica core particles (agreed to TEM and SEM, Fig. 1f). The specific increase in the maximum absorption peak can be observed in Supplementary Fig. 4b for three batches of silver nanoshells in parallel. Notably, the absorption band of silver is close to 355 nm (the wavelength for Nd:YAG laser used for LDI MS), beneficial for matrix use compared with gold nanoparticles^16, 23, 40^. The light absorbance increased and the plasmon resonance peak became broader with higher loading ratio of silver, agreed to TEM, SEM, DLS, and EDX characterizations demonstrating the adjustable properties of silver nanoshells through multi-cycled silver mirror reactions. Compared to previous literatures mostly focused on core-shell particles either with dispersed Ag nanoparticles (~4 nm) on surface^41^ or thick (~10–80 nm) densely packed shell structures^39, 42^ through surface coating techniques^39, 41–44^, our work was featured with SiO_2_@Ag affording thin (~2–10 nm), discontinuous, and tunable silver nanoshells (Fig. 1c–e).

The surface chemistry of silver nanoshells can be adjusted considering the binding affinity between thiol groups and silver^45, 46^. We employed thiol-aptamers to modify the silver nanoshells and observed the new UV-Vis absorption peak at ~254 nm for nucleic acid composites (Supplementary Fig. 8a), indicating the successful functionalization of aptamers^14, 23, 46^. We validated the surface chemistry of modified materials by Fourier transform infrared (FTIR) spectroscopy (Supplementary Fig. 8b). The appearance of typical peaks for –CH_2_– (at 2925 cm^−1^ and 2851 cm^−1^), –PO_2_ (at 1147 cm^−1^ and 1249 cm^−1^), and –C=O (at 1738 cm^−1^) confirmed the functional surface of silver nanoshells after modification with aptamers^23, 34^, which may allow target enrichment and selective detection of specific molecules.

### Selection of designer SiO_2_@Ag for LDI MS analysis

**Fig. 2:**
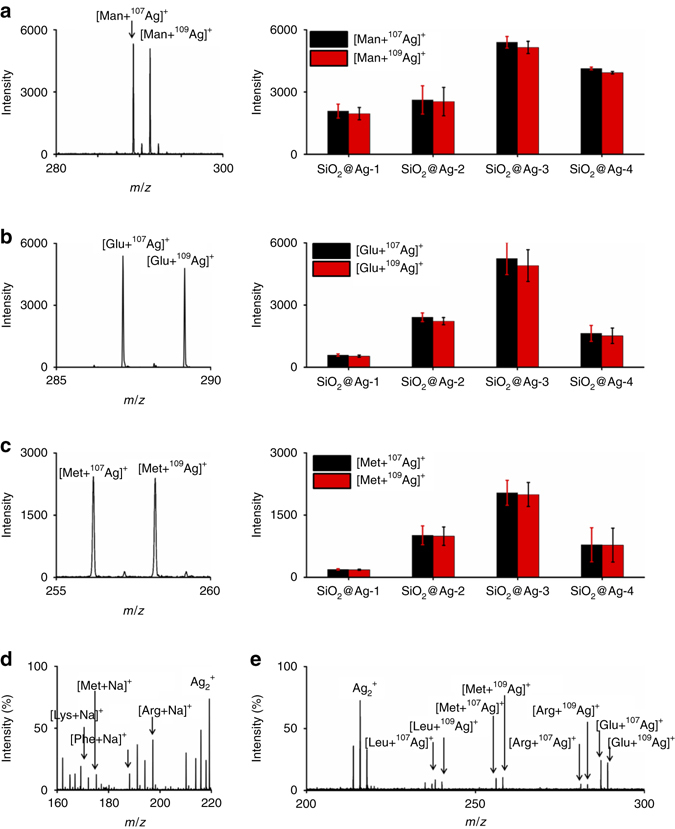
Selection of optimized SiO_2_@Ag. LDI MS analysis of 10 ng μL^−1^ **a** mannitol, **b** glucose, and **c** methionine in the positive ion mode using SiO_2_@Ag-1, SiO_2_@Ag-2, SiO_2_@Ag-3, and SiO_2_@Ag-4 as matrices, including typical mass spectra showing silver-adducted molecular peaks of theses metabolites and mean intensities of signals obtained using different SiO_2_@Ag particles as matrices. The *error bars* were calculated as s.d. of three measurements (*p* < 0.05, one-sided *t*-tests). Mass spectra of **d** 2 nM lysine, methionine, arginine, and phenylalanine in 0.5 M NaCl and **e** 2 nM leucine, methionine, arginine, and glucose in 5 mg mL^−1^ bovine serum albumin solution with a KCl concentration of 0.5 M

To select the optimized materials, we applied the SiO_2_@Ag as new matrices in analysis of small metabolites, through mixing SiO_2_@Ag particles with different analytes solutions and drying the mixture for direct LDI MS. Notably, we obtained strong silver ions-adducted molecular peaks for all the spectra in Fig. 2, distinct from matrices composed of other noble metals^6, 7, 14, 15, 19^. We observed *m*/*z* of 289.26 [M+^107^Ag]^+^ and 291.26 [M+^109^Ag]^+^ for mannitol (Fig. 2a), *m*/*z* of 287.23 [M+^107^Ag]^+^ and 289.23 [M+^109^Ag]^+^ for glucose (Fig. 2b), and *m*/*z* of 256.20 [M+^107^Ag]^+^ and 258.20 [M+^109^Ag]^+^ for methionine (Fig. 2c). The peak intensities of [M+^107^Ag]^+^ and [M+^109^Ag]^+^ were close, evidencing the even isotope abundance of silver in nature. In the production of [M+Ag]^+^ ions, competitive Ag^+^ ions can cationize molecules containing π-bonds by the Dewar model^47, 48^ and polar functional groups (like hydroxyl group) through the ion–dipole interaction^49, 50^.

Specifically, SiO_2_@Ag-3 afforded the highest mean signal intensities in triplicate experiments, superior to SiO_2_ (Supplementary Fig. 9), SiO_2_@Ag-1, SiO_2_@Ag-2, and SiO_2_@Ag-4 (*p* < 0.05, Fig. 2a–c, Supplementary Table 2). In addition, we viewed molecular peaks with Na^+^ adduction as well in Supplementary Fig. 10, including *m*/*z* of 205.28 [M+Na]^+^ for mannitol (Supplementary Fig. 10a), *m*/*z* of 203.26 [M+Na]^+^ for glucose (Supplementary Fig. 10b), and *m*/*z* of 172.21 [M+Na]^+^ for methionine (Supplementary Fig. 10c). We summarized typical *m*/*z* values of identified molecular peaks in Supplementary Table 3. In the above selection of optimized materials, the shell structure of SiO_2_@Ag was the key parameter and the differences in the physical and chemical properties of various small metabolites can lead to the different ionization efficiency as observed, even using the given SiO_2_@Ag in LDI MS analysis. SiO_2_@Ag-3 with a thin packed silver layer (Fig. 1) afforded structural stability, specific nano-gaps, and surface plasmon resonance, leading to significantly higher performance over the SiO_2_ and SiO_2_@Ag-1/2/4. Overloading of silver on the silica (SiO_2_@Ag-4) reduced the analytical efficiency of SiO_2_@Ag-3, owing to the surface aggregation of excess silver on the silica core (Fig. 1f, i; Supplementary Fig. 4b), rather than clumping of SiO_2_@Ag particles (Fig. 1g; Supplementary Fig. 11). Insufficient loading of silver (SiO_2_ and SiO_2_@Ag-1/2) also affected the analytical efficiency of SiO_2_@Ag-3 (Fig. 1e), due to more surface area from silica cores exposed to metabolites and less specific silver nano-crevices. Notably, the unique silver peaks (Ag_1-3_^+^) can be used for mass calibration in each spectrum for accurate analytes identification.

In addition to the bare silver nanoshells, surface functionalization with molecular probes can lead to specific capture of molecules. As a demonstration, we enriched kanamycin using the aptamers-functionalized silver nanoshells and detected the molecular peaks at *m*/*z* of 507.18 [M+Na]^+^, 591.16 [M+^107^Ag]^+^, and 593.16 [M+^109^Ag]^+^ at low concentrations (from 200 nM to 20 μM, Supplementary Fig. 12). These results suggested that the silver nanoshells may serve as a volatile platform for selective and sensitive of capture and detection of target molecules.

We further studied the salt tolerance and protein endurance of designer silver nanoshells (SiO_2_@Ag-3) for small metabolites detection at low abundance, which can be fundamentally important for real-case applications in complex bio-fluids. Dealing with a mixture of four small metabolites (2 nM each) in highly concentrated NaCl solution (0.5 M) in Fig. 2d, we obtained Na^+^-adducted signals at *m*/*z* of 169.26 [M+Na]^+^ for lysine, 172.21 [M+Na]^+^ for methionine, 188.22 [M+Na]^+^ for phenylalanine, and 197.25 [M+Na]^+^ for arginine. In parallel, we obtained K^+^ adducted signals in 0.5 M KCl solution (Supplementary Fig. 13). In a complex sample containing proteins (5 mg mL^−1^ BSA) and salts (KCl solution, 0.5 M), we can still observe clear silver ions-adducted signals (Fig. 2e) at *m*/*z* of 238.22 [M+^107^Ag]^+^ and 240.22 [M+^109^Ag]^+^ for leucine, 256.19 [M+^107^Ag]^+^ and 258.19 [M+^109^Ag]^+^ for methionine, 281.27 [M+^107^Ag]^+^ and 283.27 [M+^109^Ag]^+^ for arginine, and 287.22 [M+^107^Ag]^+^ and 289.22 [M+^109^Ag]^+^ for glucose, demonstrating the fine salt tolerance and multiplexity of SiO_2_@Ag-3 for practical use. The nano-crevice of silver nanoshells can trap small analytes and transfer laser energy for selective desorption/ionization process, whereas mass spectrometry served as the ideal tool to screen diverse metabolites simultaneously with high throughput.

### Diagnosis of postoperative brain infection by CSF detection

**Fig. 3:**
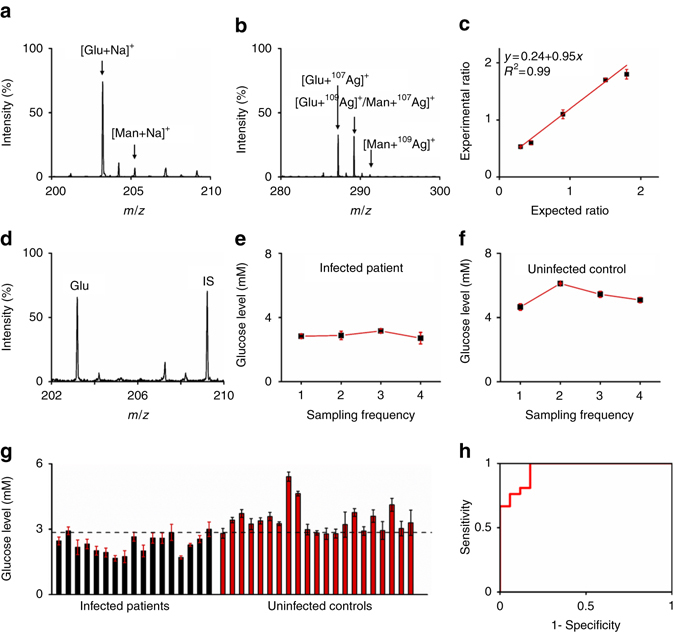
Postoperative brain infection diagnosis based on glucose quantitation. Mass spectra of 0.5 μL of native CSF showing **a** sodium-adducted and **b** silver-adducted characteristic molecular peaks of glucose and mannitol; **c** the calibration curve obtained by plotting experimental ratio of analyte/isotope (A/I) as a function of expected ratio of A/I for glucose; **d** typical mass spectrum of glucose and its isotope as internal standards (A/I, 1/1). The isotope (IS) contained six ^13^C. Glucose levels as measured in CSF from the **e** infected patient and **f** uninfected control in consecutive 4 days. **g** Glucose levels in CSF samples using the particle-assisted LDI MS and **h** the corresponding ROC curve showing the diagnostic sensitivity and specificity. The optimized threshold (2.85 mM) according to the ROC curve was denoted by the *dotted line*. Five independent experiments were performed for each sample to calculate the standard deviation (s.d.) as *error bars*. Data were shown as the mean ± s.d. (*n* = 5)

The incidence of postoperative central nervous system infection after neurosurgical procedures is ~5–7 % globally and even higher if antibiotic prophylaxis is not applied in time, which significantly endangers the human health by damaging the nerve systems^51, 52^. CSF detection is decisive to identify patients with postoperative brain infection and glucose level in CSF has been affirmed as diagnosis criteria^52, 53^. However, present measurement glucose level in CSF for diagnostics uses the traditional biochemical method, encountering many problems including tedious sample pre-treatment, long time of biochemical reaction, large sample consumption (~200 μL), and so forth. Using the designer silver nanoshells (within minutes for the overall experiments), we directly detected the Na^+^-adducted glucose at *m*/*z* of 203.26 (Fig. 3a) and Ag^+^-adducted glucose at *m*/*z* of 287.23 and 289.23 (Fig. 3b), together with mannitol (*m*/*z* of 205.28, 289.23, and 291.23) and other small metabolites (Supplementary Table 4), consuming 0.5 μL of native CSF only. Besides the accurate mass measurement, we also performed MS/MS analysis of these signals and compared these results with tandem mass spectra from standards for identification (Supplementary Figs. 14–17).

It should be mentioned that LDI MS is usually used for qualitative or at most semi-quantitative analysis, and the MS peak intensity cannot be used for quantification owing to the non-predictable analyte-dependent ion production behavior. Thus, we coupled isotopic quantification to measure the concentration of glucose for diagnostic purpose in CSF, affording consistent calibration curves with coefficient of determination (*R* ^2^) of 0.99 using isotopes with six ^13^C (Fig. 3c) and using one ^13^C (Supplementary Fig. 18). Figure 3d showed one typical mass spectrum of analyte from five experiments (Supplementary Fig. 19 showing the other four) and its isotope with analyte/isotope (A/I) ratio of 1/1. Notably, compared to the biochemical method based quantification, the sample volume was reduced from 200 to 0.5 μL and the experiment time was reduced from hours to several minutes by the LDI MS. The isotopic quantification afforded the average recovery of ~131% with coefficient of variation (CV) within 6% (Fig. 3c), which was comparable to the biochemical method that afforded the average recovery of ~107% with CV within 14% (Supplementary Fig. 20). We concluded that the silver nanoshells (SiO_2_@Ag-3)-assisted LDI MS achieved fast, sensitive, accurate, multiplex, quantitative, and reproducible detection of small metabolites in native CSF without any enrichment or purification.

We applied the silver nanoshells-assisted LDI MS to monitor the glucose level in patients CSF for diagnosis of postoperative brain infection. Different from blood systems, glucose level in CSF is more stable as an energy source for brain and has a vital role in neurotransmitter synthesis and synaptic neurotransmission^54, 55^. Postoperative brain infection causes low glucose levels in CSF due to the metabolism of operation introduced bacteria^53, 56^, and the threshold of glucose level is ~2.8–3.9 mM in continued drained CSF as demonstrated in the earlier reports^56, 57^. As displayed in Fig. 3e for a patient with brain infection, the glucose levels were 2.83, 2.88, 3.16, and 2.71 mM for consecutive 4 days after an operation, suggesting postoperative brain infection. For comparison, the as measured glucose levels of a patient without infection were 4.66, 6.12, 5.45, and 5.09 mM using silver nanoshells-assisted LDI MS also for consecutive 4 days (Fig. 3f). Further, we detected 17 CSF samples from infected patients and 21 CSF samples from uninfected controls by our approach (Fig. 3g), which showed consistence with the current biochemical method used in hospitals affording coefficient of determination (*R* ^2^) of 0.92 (Supplementary Fig. 21, see also Supplementary Table 5 for details). We obtained the optimized diagnostic sensitivity of 81.6% with specificity of 88.2% based on the cut-off value of 2.85 mM according to the receiver operating characteristic (ROC) curve (area under curve of ROC (AUC) of 0.961, Fig. 3h). Previous LDI MS approaches were mostly limited to standard analysis, spiked experiments, or imaging tests, considering the difficulties in sample pre-treatment of complex bio-fluids for enrichment and purification. In the above demonstration, we demonstrated clinical diagnosis of patients with postoperative brain infection as well as daily monitoring by advanced LDI MS analysis of CSF, using the silver nanoshells with designer structural parameters.

### Pharmacokinetic study in serum and CSF during drug treatment

**Fig. 4:**
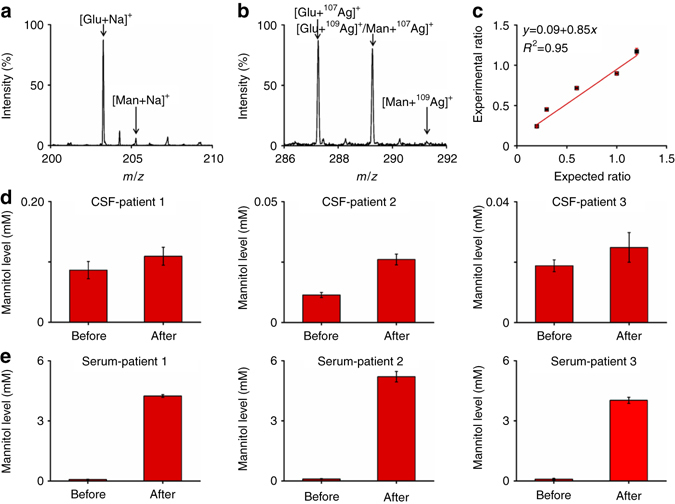
Pharmacokinetic study based on mannitol detection in bio-fluids. Mass spectra of 0.5 μL of native serum showing **a** sodium-adducted and **b** silver-adducted characteristic molecular peaks of glucose and mannitol; **c** the calibration curve obtained by plotting experimental ratio of analyte/isotope (A/I) as a function of expected ratio of A/I for mannitol; mannitol levels as measured in **d** CSF and **e** serum from three patients before and 30 min after intravenous injection (*p* < 0.05, Wilcoxon signed-rank test). Five independent experiments were performed for each sample to calculate the standard deviation (s.d.) as *error bars*. Data were shown as the mean ± s.d. (*n* = 5)

Pharmacokinetic study guides the overall therapeutics and has been applied universally in clinics during diverse medical treatment. It is instrumental to understand the distribution and metabolism of selected drugs for pharmacokinetic study. For instance, mannitol, a small metabolite, has been long employed for reducing intracranial pressure, and increasing cerebral perfusion and blood flow during clinical treatments^58, 59^. Nevertheless, monitoring concentrations of small metabolites/drugs (e.g., mannitol) in serum and CSF is difficult by traditional electrospray ionization (ESI) MS techniques due to the high samples complexity and low molecular abundance, which requires enrichment or separation (such as, chemo-selective extraction and liquid/gas chromatography) and is time-consuming, labor-intensive, and very expensive for large-scale use^60–62^. With 0.5 μL of native serum, we directly obtained the Na^+^-adducted mannitol at *m*/*z* of 205.28 (Fig. 4a) and Ag^+^-adducted mannitol at *m*/*z* of 289.23 and 291.23 (Fig. 4b), together with glucose (*m*/*z* of 203.26, 287.23, and 289.23) and other small metabolite (Supplementary Table 6), based on the optimized silver nanoshells. We compared tandem mass spectra of these signals from bio-samples and standards, in addition to the accurate mass measurement for identification (Supplementary Figs. 14–17). As control experiments, we observed no signals by LDI MS without any matrix due to low LDI efficiency (Supplementary Fig. 22a). We obtained overwhelming background noises with few peaks from small metabolites using organic matrix (e.g., α-cyano-4-hydroxycinnamic acid, CHCA) in Supplementary Fig. 22b and can only recognize glucose signal using inorganic matrix (gold nanoparticles) in Supplementary Fig. 22c, both of which demonstrated the advantages of silver nanoshells over current matrices in bio-analysis.

For diagnostic purpose, we also applied isotopic quantification to measure the concentration of mannitol in serum. The calibration curve afforded a coefficient of determination (*R* ^2^) of 0.95 in a dynamic range of 100–600 ng μL^−1^ (Fig. 4c) and high reproducibility in serum detection with a standard deviation of 1.311% for five independent tests (Supplementary Fig. 23). We also examined the stability of silver nanoshells for serum detection and found that SiO_2_@Ag can be capable of LDI MS analysis for at least 5 months (Supplementary Fig. 24). Notably, despite the high sample complexity of serum, we achieved efficient LDI MS analysis of small metabolites without any sample pre-treatment similar to that in CSF detection and validated the performance of the silver nanoshells (SiO_2_@Ag-3) in complex bio-fluids.

We monitored the concentration changes of mannitol in serum and CSF, and demonstrated distribution of mannitol in blood and CSF systems during therapeutic treatment of three patients with cerebral edema. Notably, the blood and brain/CSF systems are divided by the blood–brain/CSF-barriers, which can only allow specific molecules to pass^63–65^. In CSF samples analysis of three patients based on the silver nanoshells-assisted LDI MS, mannitol concentrations showed minor increase by ~29.41–128.96% before and 30 min after intravenous injection during the therapeutic treatment (Fig. 4d; Supplementary Table 7, *p* < 0.05). In contrast, the mannitol concentrations after drug delivery were significantly higher than untreated ones, showing ~45.23–53.35-folds increase in serum samples of the patients (Fig. 4e; Supplementary Fig. 25; Supplementary Table 7, *p* < 0.05). The blood–brain/CSF-barriers are fundamentally important to maintain the normal physiological activity of living systems, whereas the molecular permeability of the barrier guides the use of drugs in clinical practice^63–65^. Our results demonstrated the application of silver nanoshells as matrix for LDI MS to investigate the function of blood–brain/CSF-barriers in dividing blood and brain/CSF systems, as well as the permeability enabling the pass of mannitol^66, 67^. Considering the advantages of silver nanoshells-assisted LDI MS over ESI MS that required sample pre-treatment^61, 62^ and LDI MS using other matrices (Supplementary Fig. 22), we anticipate our approach to engage the advanced pharmacokinetic study of various small metabolites and drugs for large-scale clinical use.

## Discussion

In summary, we introduced silver nanoshells (SiO_2_@Ag) as matrices for direct LDI MS detection of small metabolites in bio-fluids, and further developed a platform technology for metabolic analysis-based disease detection and therapeutic evaluation. We synthesized series of silver nanoshells with controlled structures by multi-cycled silver mirror reactions and selected the designer silver nanoshells with optimized analytical performance. We revealed the mechanism for efficient LDI process associated with structural parameters of materials, and demonstrated the application in complex bio-fluids in real cases. Furthermore, coupling with isotopic quantification of selected metabolites (e.g., glucose and mannitol), we not only identified patients with postoperative brain infection by CSF analysis, but also monitored the drug concentrations in both CSF and serum to investigate the blood–brain/CSF-barriers and for pharmacokinetics study. Our work contributes to the design of materials for high-performance metabolic analysis towards precision medicine and initiates the development of diverse advanced diagnostic tools involving various metabolic biomarkers.

## Methods

### Chemicals and reagents

Ammonium hydroxide (28–30%), tetraethyl orthosilicate (TEOS, 96%), ethanol absolute (99.7%), silver nitrate (99.5%), sodium chloride (99.5%), potassium chloride (99.5%), and trifluoroacetic acid (TFA, 99%) were purchased from Sinopharm Chemical Reagent Beijing Co., Ltd (Beijing, China). Acetonitrile (ACN, 99%), α-cyano-4-hydroxycinnamic acid (CHCA, 99%), dithiothreitol (DTT, 99%), cetyltrimethylammonium chloride (CTAC, 99%), albumin from bovine serum (BSA, 98%), polyvinylpyrrolidone (PVP, MW = 40,000), D-glucose (99.5%), L-methionine (99%), D-mannitol (99%), L-lactic acid (98%), L-arginine (99.5%), L-tryptophan (98%), uric acid (99%), and DL-phenylalanine (99%) were purchased from Sigma, USA. Tris base and phosphate-buffered saline (PBS, 10×, pH 7.4, cell-culture grade) were purchased from Yeason Biotechnology Co., Ltd (Shanghai, China). The isotopic internal standards of glucose (labeled with six ^13^C or one ^13^C) and mannitol (labeled with one ^13^C) were purchased from Cambridge Isotope Laboratories (CIL, USA). Thiol-aptamers were obtained from Sangon Biotech Co., Ltd. (Shanghai, China). All aqueous solutions were prepared using deionized water (18.2 MΩ cm, Milli-Q, Millipore, GmbH) throughout the experiments.

### Synthesis of materials

SiO_2_@Ag core-shell particles were prepared through controlled surface coating of silver on silica nanoparticles. Smooth and uniform silica nanoparticles were synthesized as the hard templates using the Stöber method^68^. To perform the surface coating, the silver mirror reaction was used. Overall, 1.80 g of the prepared silica nanoparticles was dispersed in 90 mL of ethanol solution. In a typical reaction cycle, freshly prepared [Ag(NH_3_)_2_]^+^ ion solution (0.59 M, 10 mL) was quickly added to the above particles dispersion and sonicated at room temperature for 30 min. Then the particles were mixed with 300 mL of PVP ethanol solution (0.5 mM). During the experiment, PVP was employed as both the stabilizer and reductant^38^. The mixtures were stirred at 30/50/70 °C for 2/4/5/7 h for the formation of silver nanoshells (SiO_2_@Ag). The structures of silver nanoshells can be adjusted by conducting the above reactions towards at the optimized condition (at 70 °C for 7 h, further increased temperature or time may cause unwanted precipitation) for 1–4 rounds to obtain SiO_2_@Ag-1/2/3/4. The resulting products were washed with 50 mL of ethanol and deionized water and centrifuged at 10,000×*g* for 10 min for three times. The final SiO_2_@Ag particles were dried at 60 °C and stored as powders. The UV-Vis spectra of particles were checked and the qualified particles would show the absorption peak at ~436 nm without red shift (suggesting no overloading of silver).

Gold nanoparticles were synthesized using the in-solution seeding approach^16^. The seeding solution was firstly prepared by vigorous mixing of 10 mL of aqueous CTAC solution (0.1 M) and 515 mL of HAuCl_4_ (4.86 mM) with 450 mL of NaBH_4_ solution. Then the solution was aged for at least 1 h in a hot bath and then was diluted for ten times. Next, 10 mL of CTAC solution (0.1 M) was mixed with 515 mL of HAuCl_4_ (4.86 mM) and 75 mL of ascorbic acid (0.04 M). Overall, 100 mL of diluted seed solution was added into the above mixed solution under sonication and kept in darkness for 2 days to obtain the final gold nanoparticles after centrifugation and washing with water.

### Surface functionalization

To modify silver nanoshells with aptamers, we used a post-grafting method^23, 46^. Briefly, 48 μL of PBS buffer was added into 1 OD of SH-modified kanamycin aptamers (5′-SH-(CH_2_)_6_-TGGGGGTTGAGGCTAAGCCGA-3′) to a final concentration of 100 μM. 0.48 μL of DTT solution (10 μM) was mixed with aptamer solution and incubated at 37 °C for 1 h. Then 200 μL of SiO_2_@Ag-3 (0.5 mg mL^−1^) was added to the system and gently shaken at room temperature for 24 h with addition of NaCl (0.1 M) after 12 h. After incubation, the mixture was centrifuged for 30 min at 13,000×*g* and re-dispersed in PBS.

### Material characterization methods

TEM images, HRTEM images, and elemental mapping images were collected using a JEOL JEM-2100F instrument. Normally, ~8–10 μL of ethanol suspension of materials was deposited onto a copper grid before observation. During sample preparation for elemental mapping, 1 mg mL^−1^ of water dispersions of SiO_2_@Ag-3 were mixed with 10 μg μL^−1^ glucose (1/1, V/V) for 30 min and supernatant was discarded by centrifugation at 10,000×g for 10 min. The precipitates were washed and re-dispersed in water and put onto micro-grids for analysis. The inter planar space was measured by Digital Micrograph version 2.5 software (Gatan). SEM images and EDX spectra were recorded on Hitachi S-4800 by dropping the materials suspensions on the aluminum foil. Room temperature optical absorption spectra of the materials were obtained on an AuCy UV1900 spectrophotometer. Fourier transform infrared spectroscopy (FTIR) was performed on Nicolet iN10 MX (Thermo Scientific, USA). Nitrogen adsorption isotherms were obtained on Micromeritics ASAP 2020 M and the samples were degassed in vacuum before tests. Zeta potential and dynamic light scattering size measurements were performed on a Nano-ZS90 instrument in water at 25 °C (Malvern, Worcestershire, UK).

### Mass spectrometry analysis

For LDI MS detection of metabolites, standard small molecules (glucose, mannitol, methionine, tryptophan, uric acid, lactic acid, arginine, and phenylalanine) were dissolved in deionized water by step-wise dilutions with the concentration ranging from 1 μg μL^−1^ to 1 ng μL^−1^. Standard molecules were mixed with salts (NaCl, KCl, 0.5 M) and proteins (BSA, 5 mg mL^−1^) to explore the detection efficiency in complex samples. In a typical LDI MS experiment, SiO_2_@Ag particles and gold nanoparticles were dispersed in water at a concentration of 0.5 mg mL^−1^ for matrix use. CHCA was dissolved in 0.1% TFA buffer (water/ACN, 50/50, v/v) at a concentration of 4 mg mL^−1^. Then 500 nL of matrix slurry was deposited with 500 nL of analytes solution on a stainless steel target plate and dried for LDI MS analysis. Mass spectra were collected in the reflection mode employing delayed extraction on 5800 Proteomics Analyzer (Applied Biosystems, Framingham, MA, USA) with the Nd:YAG laser (1 kHz 355 nm). The repetition rate and an acceleration voltage were set as 200 Hz and 20 kV, respectively. The delay time for this experiment was optimized to 200 ns. The number of laser shots was 200 per analysis for all LDI MS experiments. Only MS signals with signal-to-noise ratio over 10 were used for identification of molecules. Mass calibration was conducted by background substrate mass peaks of [Ag]_*n*_^+^ (*n* = 1–3) in each spectrum for accurate mass measurement. MS/MS of selected molecular peaks of glucose and mannitol (from bio-samples and standards) were performed and compared for identification purpose in addition to the accurate mass measurement. The mass accuracy was within 50 ppm, which was comparable to previous reports^69^. No smoothing procedures were applied and all spectra were directly used for analysis.

For enrichment and detection of kanamycin, 50 μL of aptamers modified silver nanoshells slurry was centrifuged and the precipitate was re-dispersed in tris–HCl buffer (pH 7.5, 20 mM). Overall, 1 mL of kanamycin with different concentrations (from 200 nM to 20 μM) was mixed with the dispersion above and incubated at 37 °C for 1 h. The mixture was centrifuged to discard supernatant and re-dispersed in 0.5 μL of 0.1% TFA buffer (water/ACN, 20/80, v/v) for LDI MS analysis as established above.

### Isotopic quantification

The isotopes of selected metabolites were introduced as the internal standard for quantification use^17^. Standard solutions of small molecules with different concentrations were employed to plot the calibration curves. The isotopes were dissolved in water with concentrations of 250 ng μL^−1^ and 100 ng μL^−1^ for glucose and mannitol, respectively. Typically, the isotope solution was mixed with analyte solution with a volume ratio of 1:1. After dropping 500 nL of mixture solution on the plate, 500 nL of matrix solution was deposited onto it and dried for LDI MS analysis. The influence of naturally occurring ^13^C (^12^C/^13^C, 98.89%/1.11%) was eliminated by subtracting corresponding signal intensity during the quantification process using isotopes with one ^13^C. For quantification purpose, the relative intensity ratio of analytes/isotopes from five independent experiments was recorded with data shown as the mean ± s.d. (*n* = 5).

### Biochemical colorimetric method

Glucose level in 200 μL of native cerebrospinal fluid samples were detected using the traditional biochemical approach by the hexokinase/glucose-6-phosphate dehydrogenase assay (Abbott Diagnostics, Maidenhead, UK), where the glucose concentrations were obtained after enzymatic catalysis of glucose according to the colorimetric method.

### Statistical analysis

All statistical analysis in this work (including materials characterizations and bio-analytical results) was performed based on the SPSS software (version 19.0, SPSS Inc., Chicago) to calculate the *p*-value for statistical demonstration, including independent sample *t*-test, paired *t*-test, one-sided *t*-test, and Wilcoxon signed-rank test.

### Diagnosis of brain infection

CSF samples were donated by patients in Children’s Hospital of Shanghai. The CSF samples for brain infection monitoring were collected in four consecutive days during operation. For diagnostic application, 17 CSF samples were obtained from infected patients and 21 samples were obtained from uninfected controls. All the investigation protocols in this study were approved by the institutional ethics committees of the Children’s Hospital of Shanghai and School of Biomedical Engineering, SJTU. Informed consents from patients had been obtained since the project started. All samples were harvested in tubes by continued drainage without tedious lumbar punctures according to the standard clinical procedures and stored at −80 °C until use^9, 17, 70^. The CSF samples were directly detected by the LDI MS using the silver nanoshells as matrix following the previous protocols in standard analysis and five independent experiments were performed with data shown as the mean ± s.d. (*n* = 5) for diagnostic use.

### Pharmacokinetic study

For pharmacokinetic study, both blood and CSF samples were donated by three patients with cerebral edema before and 30 min after rapid intravenous injection of mannitol (20% infusion solution, Shandong Qidu Pharmaceutical Co., Ltd., China) during a surgical operation. All of the investigation protocols in this study were approved by the institutional ethics committees of the Children’s Hospital of Shanghai and School of Biomedical Engineering, SJTU. Informed consents from patients had been obtained since the project started. The harvesting of serum samples was completed by vein blood draws based on the established method^9^, whereas the sampling of CSF was the same as brain infection diagnosis experiments. The serum and CSF samples were directly detected by LDI MS using the silver nanoshells as matrix following the previous protocols in standard analysis and five independent experiments were performed with data shown as the mean ± s.d. (*n* = 5) for pharmacokinetic study.

### Data availability

The data that support the findings of this study are available from the corresponding author upon reasonable request.

## Electronic supplementary material

Supplementary Information
