# Supplementary material for: Plasmonic silver nanoshells for drug and metabolite detection
Source: Nat Commun. 2017 Aug 9;8:220. doi: 10.1038/s41467-017-00220-4 (PMC5548796; doi:10.1038/s41467-017-00220-4)
Supplement: Supplementary file 1 — Supplementary Information [file 41467_2017_220_MOESM1_ESM.pdf]

File Name: Supplementary Information
Description: Supplementary Figures and Supplementary Tables

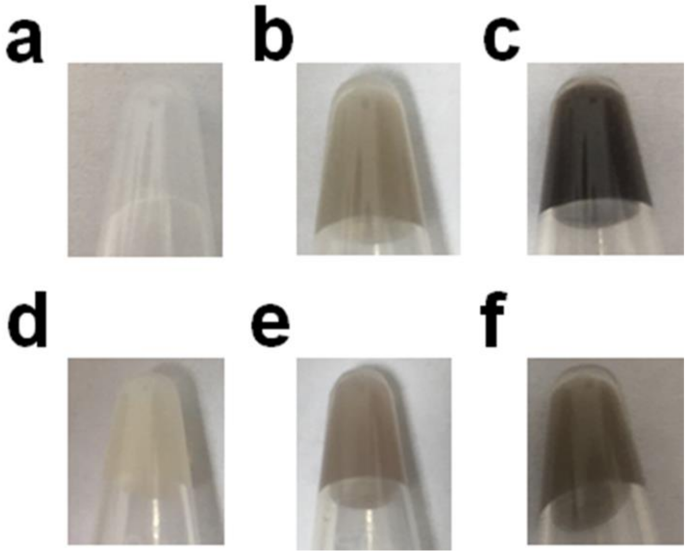


**Supplementary Fig. 1. Synthesis conditions for $SiO_2$@Ag.** Digital images recorded of $SiO_2$@Ag reaction mixtures at a) 30 °C, b) 50 °C, and c) 70 °C for 7 h and at 70 °C for d) 2 h, e) 4 h, and f) 5 h.

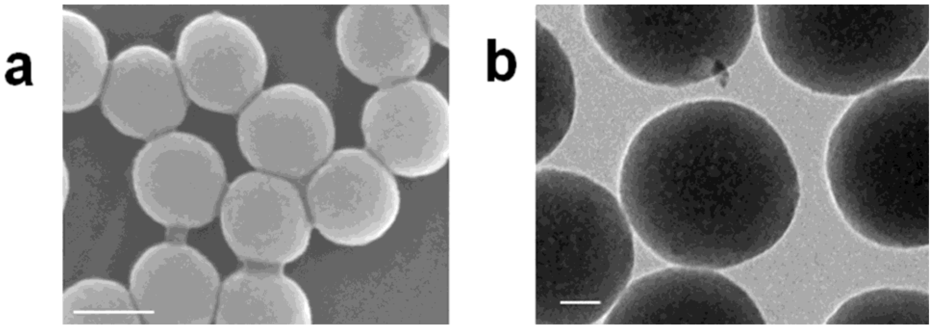


**Supplementary Fig. 2. Electron microscopy images of pure silica.** a) SEM and b) TEM images of bare $SiO_2$ particles. Scale bars: a) 150 nm and b) 50 nm.

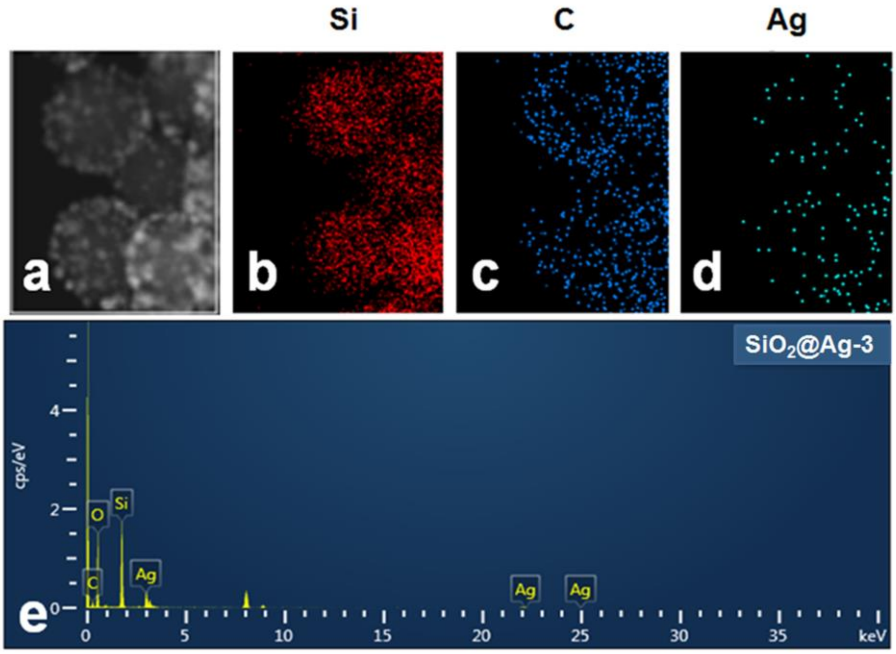


**Supplementary Fig. 3. Elemental mapping results.** a) scanning transmission electron microscopy (STEM) image and b) silicon, c) carbon, d) silver mappings, and e) typical EDX spectra for nanoshells-metabolites hybrids.

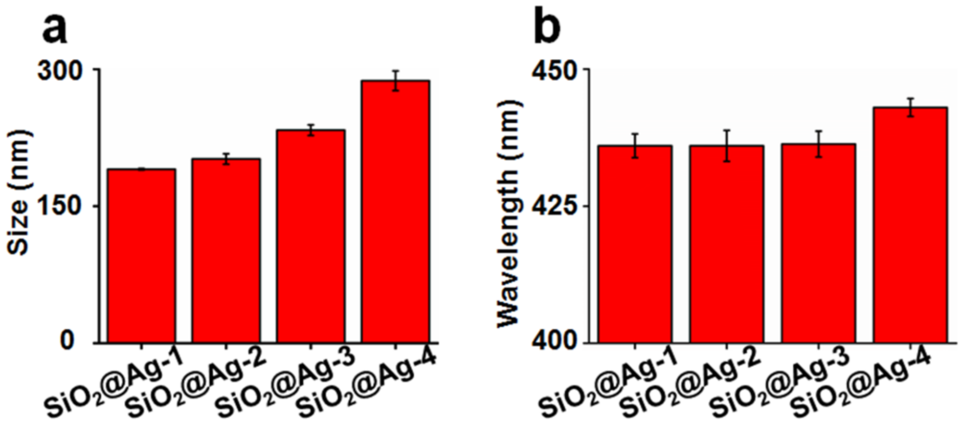


**Supplementary Fig. 4. Synthesis reproducibility.** a) Average sizes by DLS and b) wavelengths of UV-Vis absorption peaks for three batches of $SiO_2$@Ag-1/2/3/4 particles. 3 independent experiments were performed to calculate the standard deviation (s.d.) as error bars. Data were shown as the mean ± s.d. (n=3).

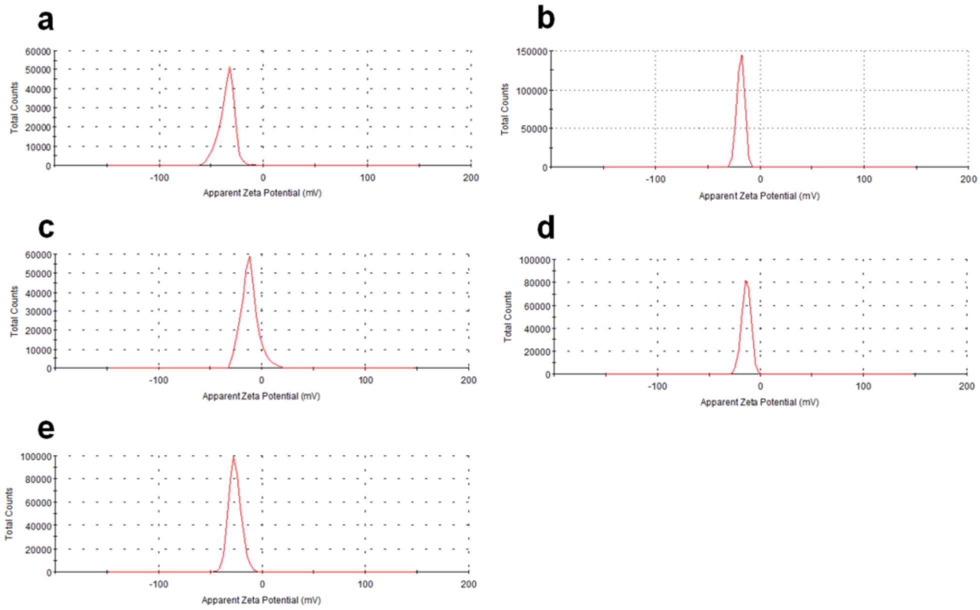


**Supplementary Fig. 5. Typical zeta potential distributions of materials.** a) $SiO_2$, b) $SiO_2$@Ag-1, c) $SiO_2$@Ag-2, d) $SiO_2$@Ag-3, and e) $SiO_2$@Ag-4.

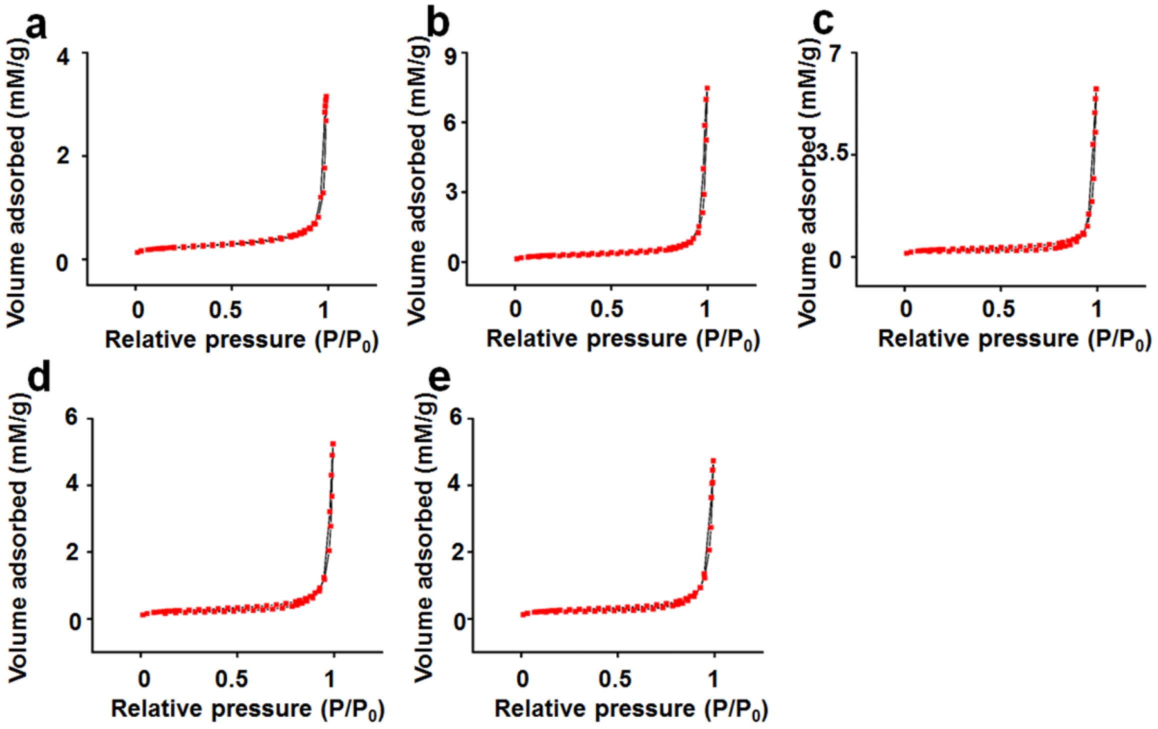


**Supplementary Fig. 6. Typical nitrogen adsorption isotherms of materials.** a) $SiO_2$, b) $SiO_2$@Ag-1, c) $SiO_2$@Ag-2, d) $SiO_2$@Ag-3, and e) $SiO_2$@Ag-4.

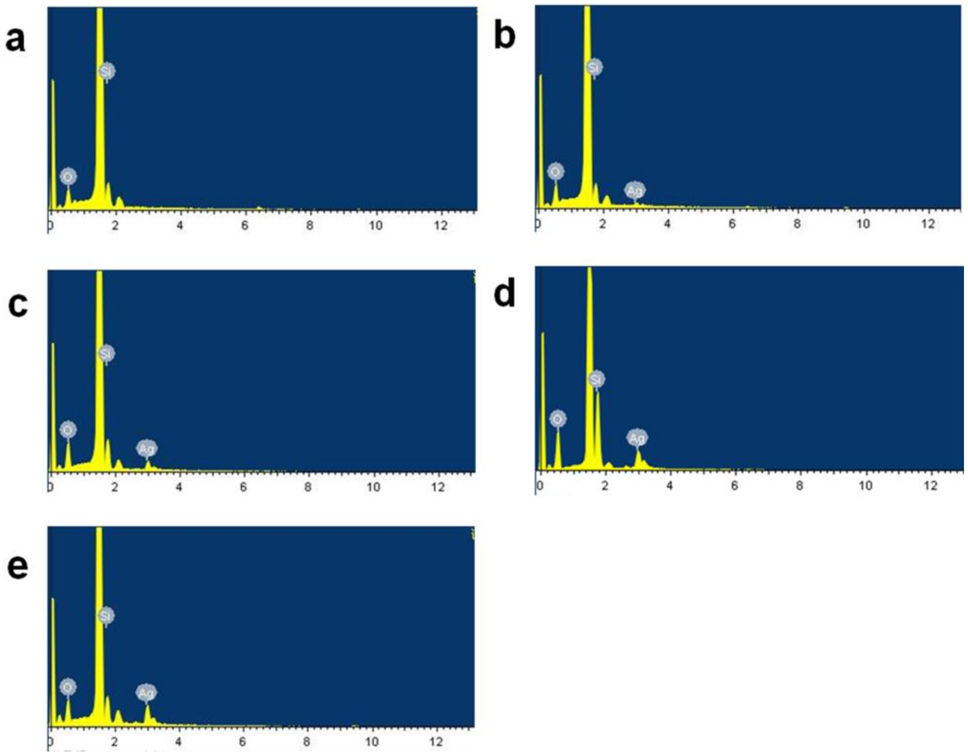


**Supplementary Fig. 7. Typical EDX spectra of materials.** a) $SiO_2$, b) $SiO_2$@Ag-1, c) $SiO_2$@Ag-2, d) $SiO_2$@Ag-3, and e) $SiO_2$@Ag-4.

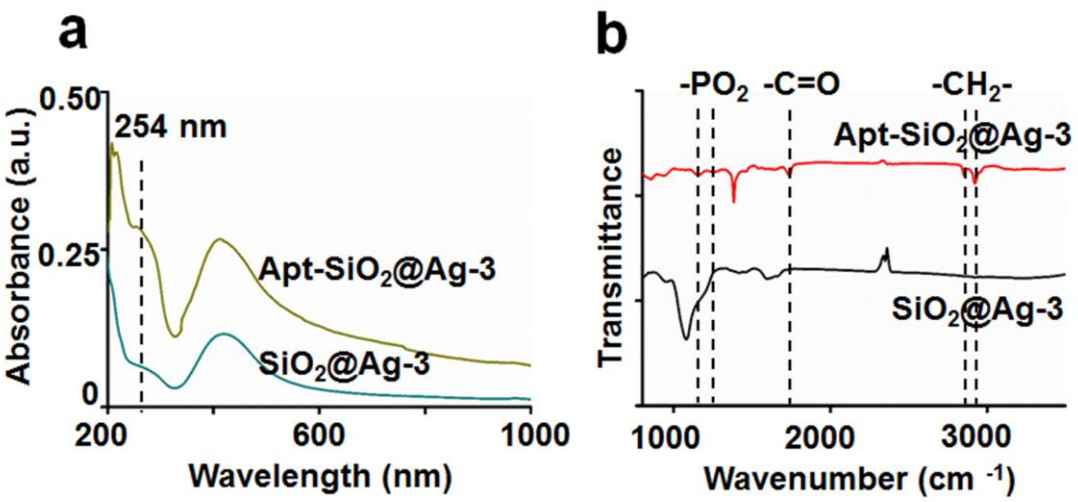


**Supplementary Fig. 8. Surface modification of silver nanoshells.** a) UV-Vis absorption spectra and b) FTIR spectra of $SiO_2$@Ag-3 and aptamers modified $SiO_2$@Ag-3 (denoted as Apt-$SiO_2$@Ag-3).

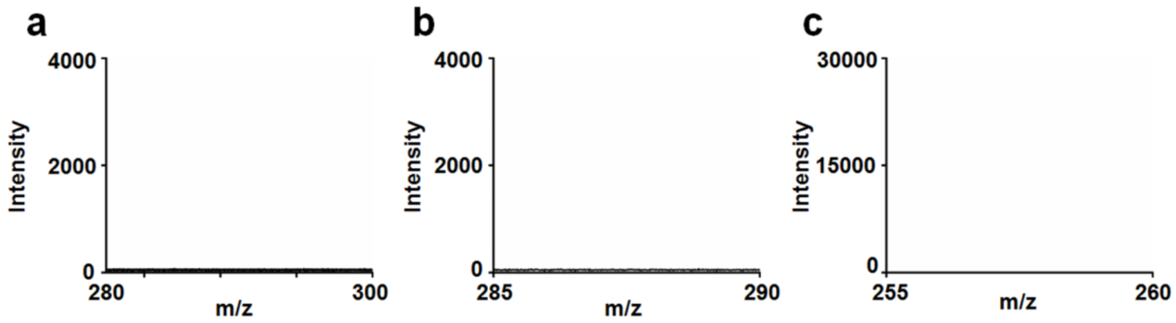


**Supplementary Fig. 9. Detection of small molecules using bare silica.** Mass spectra of 10 ng μ$L^{-1}$ a) mannitol, b) glucose, and c) methionine in the positive ion mode using bare $SiO_2$ particles as matrix.

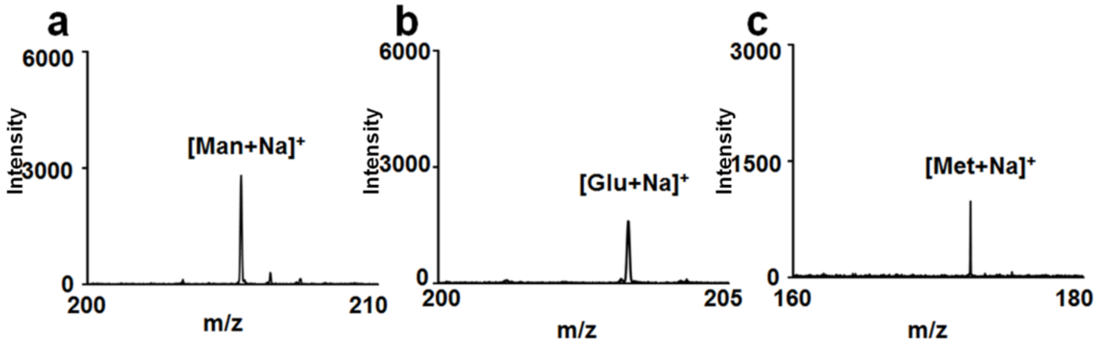


**Supplementary Fig. 10. Detection of small molecules using silver nanoshells.** Mass spectra of 10 ng μ$L^{-1}$ a) mannitol, b) glucose, and c) methionine in the positive ion mode with sodium adducted molecular peaks using $SiO_2$@Ag-3 as matrix.

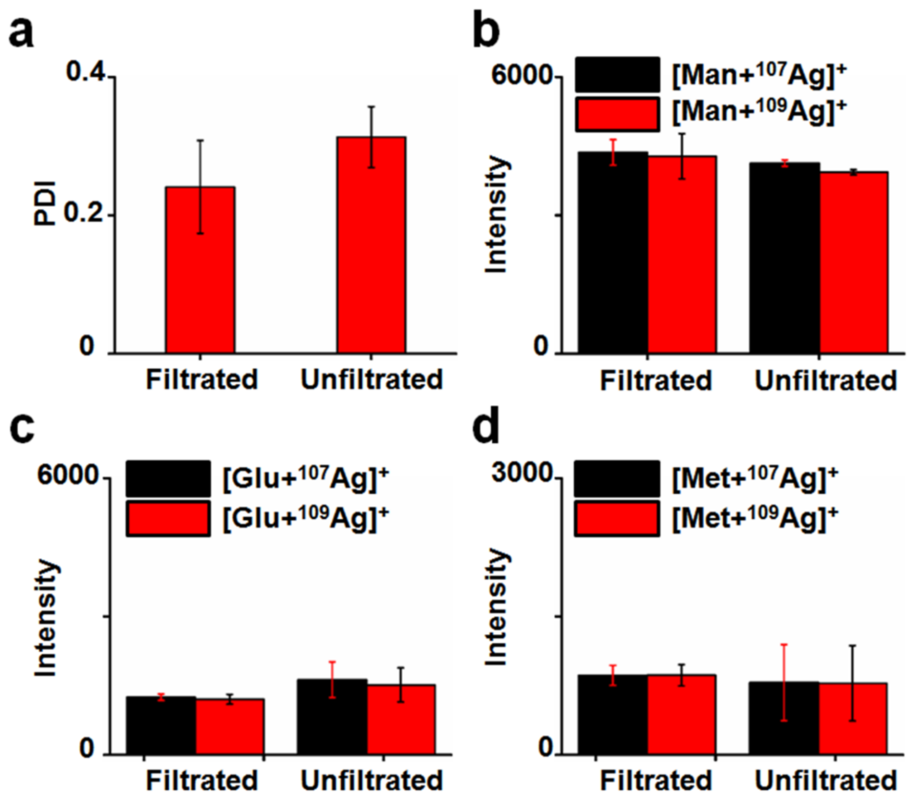


**Supplementary Fig. 11. Filtration of particles for use.** a) PDI values of $SiO_2$@Ag-4 before and after filtration by polymer membranes (pore size of 450 nm). Mean intensities of signals for 10 ng μ$L^{-1}$ b) mannitol, c) glucose, and d) methionine in the positive ion mode using $SiO_2$@Ag-4 before and after filtration by polymer membranes (pore size of 450 nm). 3 independent experiments were performed to calculate the standard deviation (s.d.) as error bars. Data were shown as the mean ± s.d. (n=3). The concentrations of all matrices were set as 0.5 mg $mL^{-1}$.

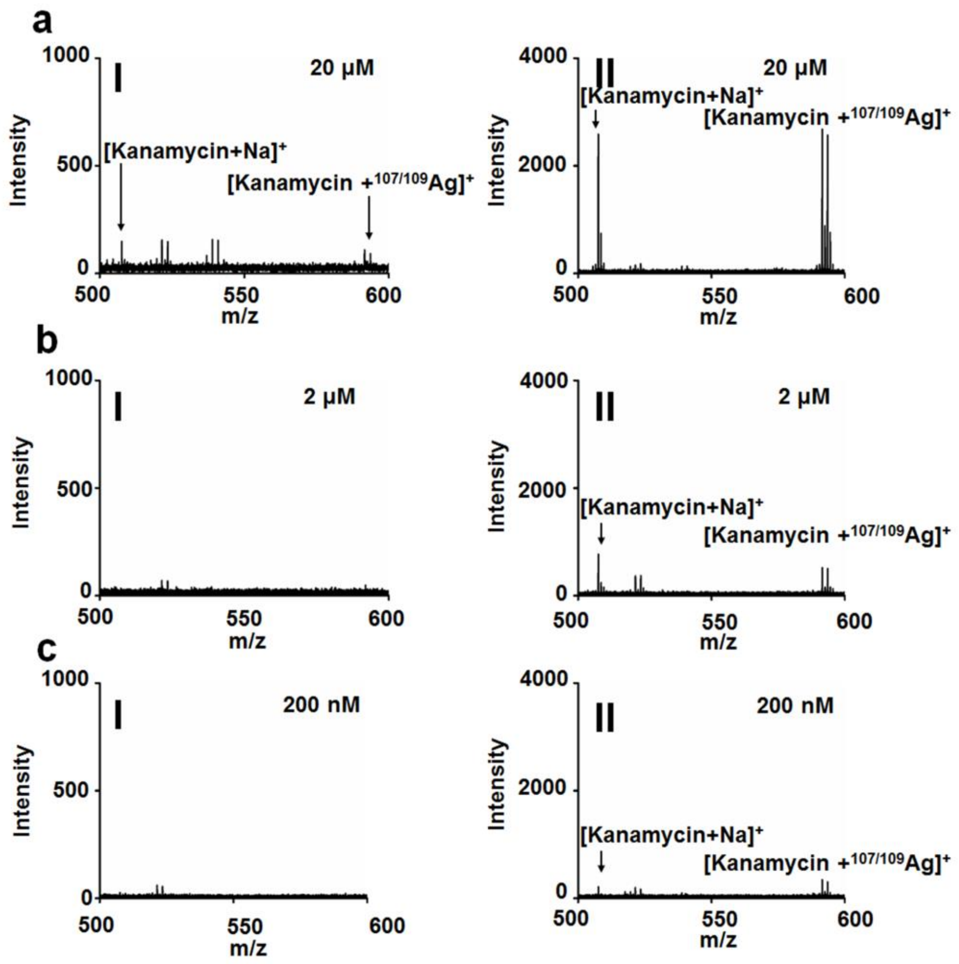


**Supplementary Fig. 12. Enrichment and detection of kanamycin.** Mass spectra of kanamycin at a) 20 µM, b) 2 µM, and c) 200 nM, I) before and II) after enrichment by Apt-$SiO_2$@Ag-3.

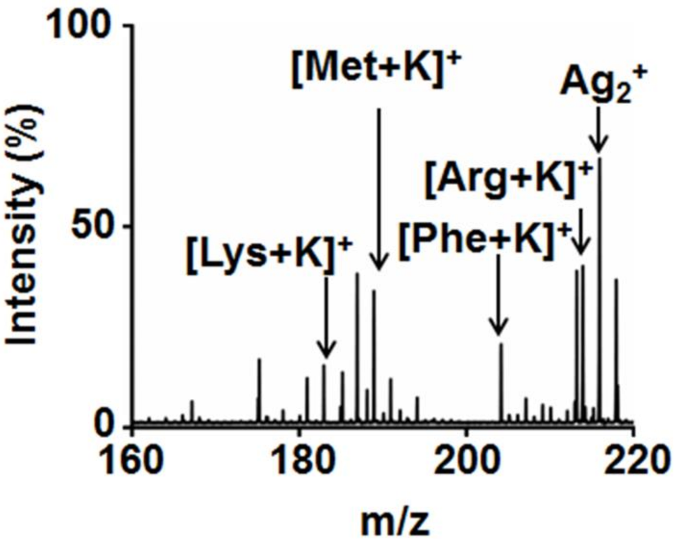


**Supplementary Fig. 13. Salt tolerance of silver nanoshells.** Mass spectra of 2 nM lysine, methionine, arginine, and phenylalanine in 0.5 M KCl using $SiO_2$@Ag-3.

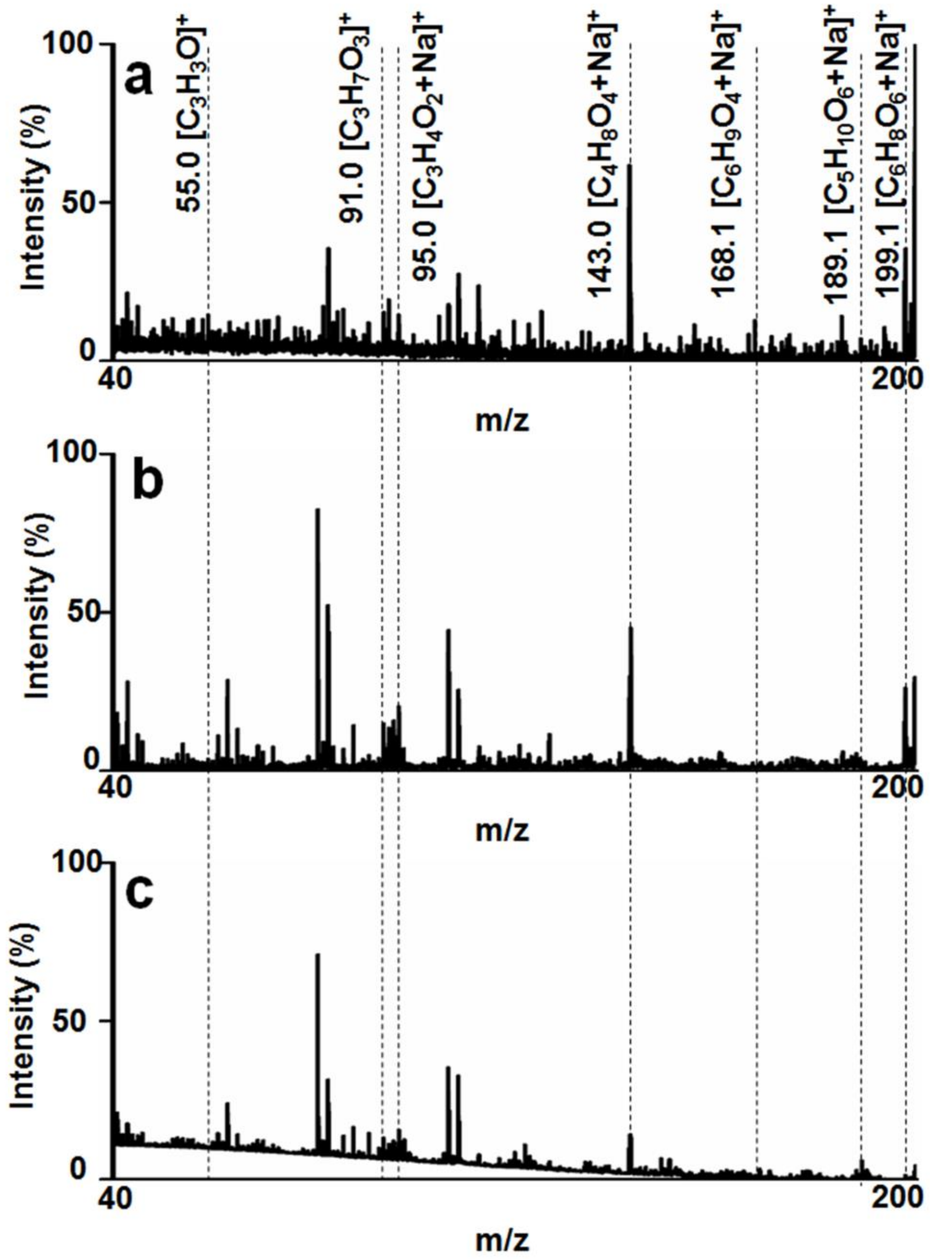


**Supplementary Fig. 14. Tandem mass spectra of sodium adducted glucose.** MS/MS of glucose at m/z of 203.2 for $[M+Na]^+$ in a) standard sample, b) CSF, and c) serum.

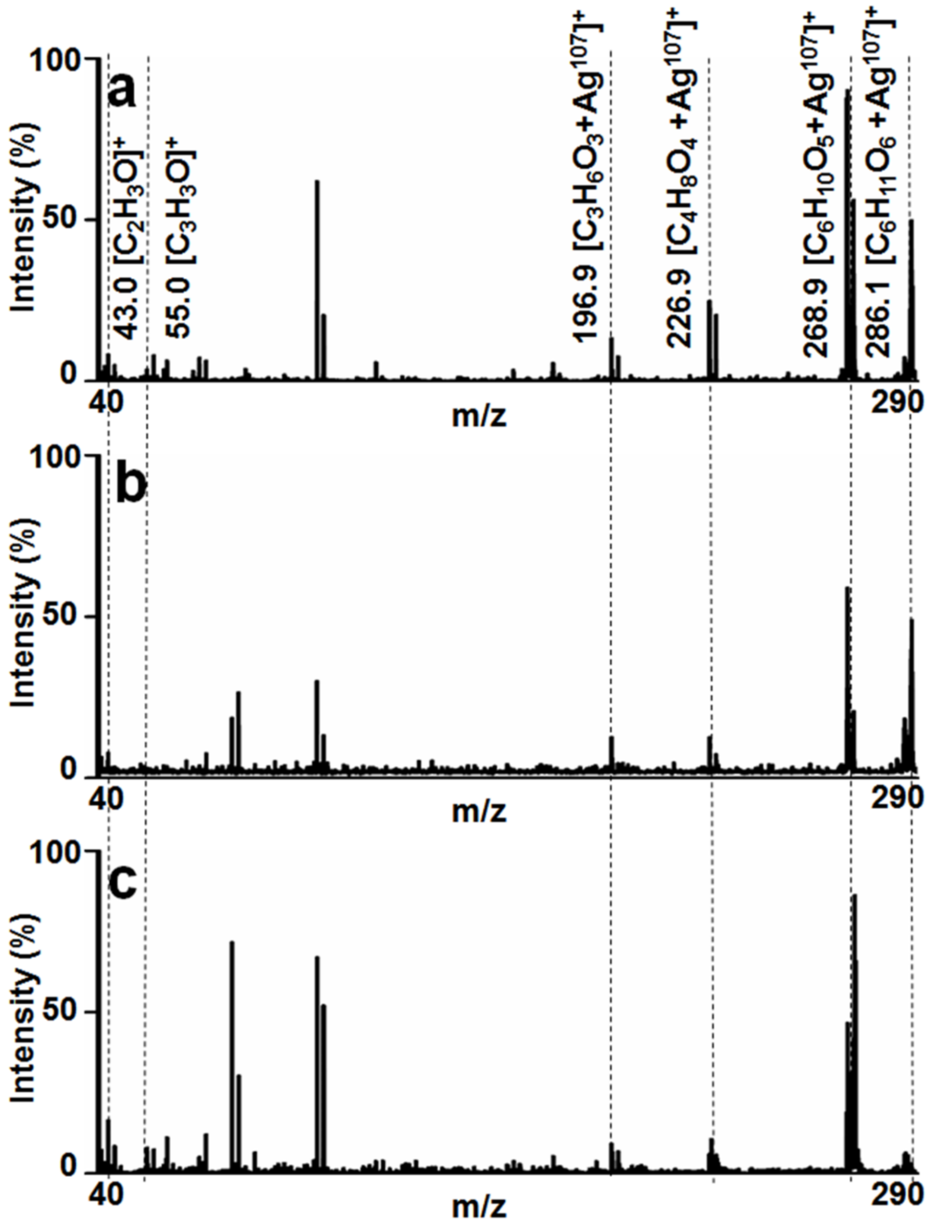


**Supplementary Fig. 15. Tandem mass spectra of silver adducted glucose.** MS/MS of glucose at m/z of 287.2 for $[M+^{107}Ag]^+$ in a) standard sample, b) CSF, and c) serum.

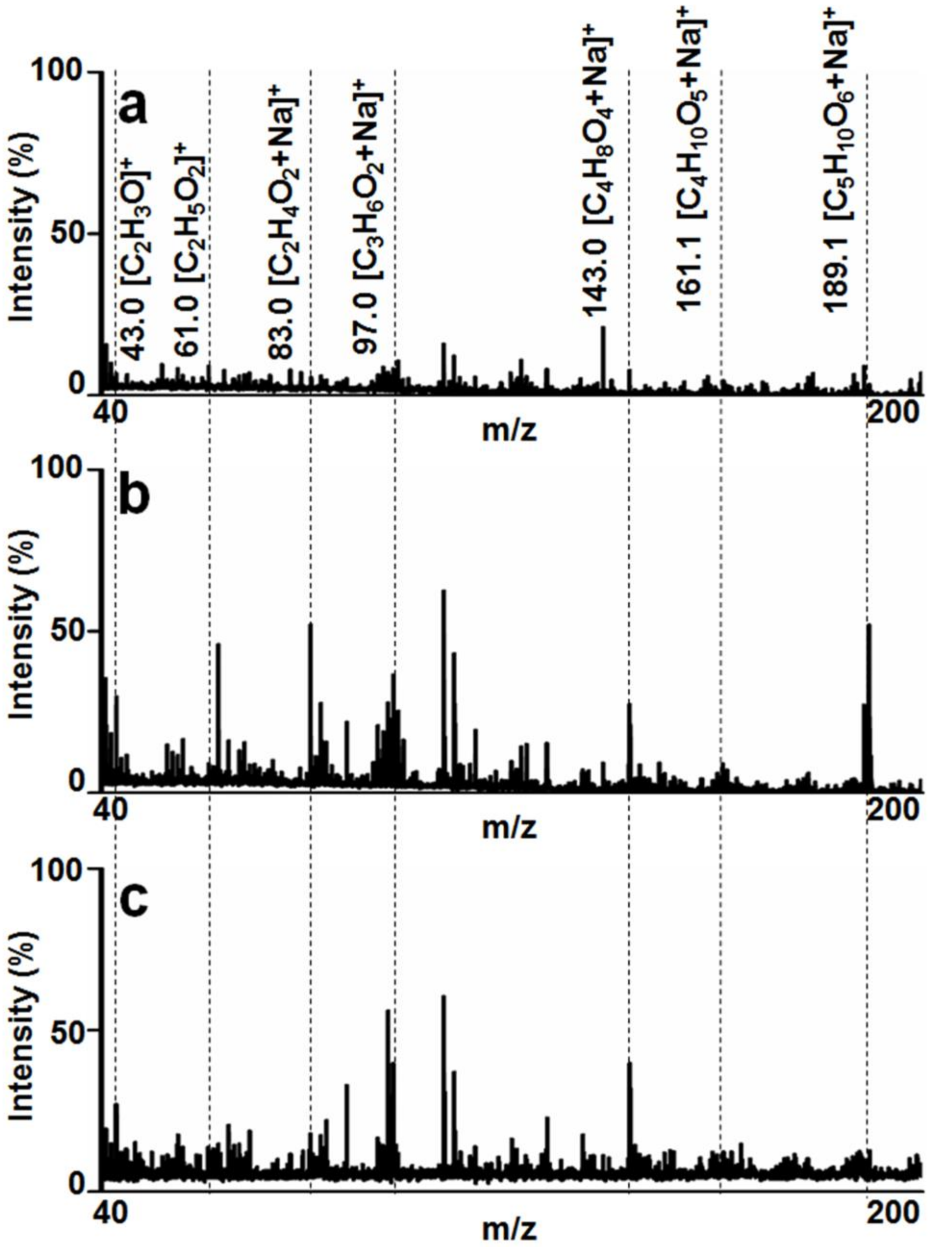


**Supplementary Fig. 16. Tandem mass spectra of sodium adducted mannitol.** MS/MS spectra of mannitol at m/z of 205.2 for $[M+Na]^+$ in a) standard sample, b) CSF, and c) serum.

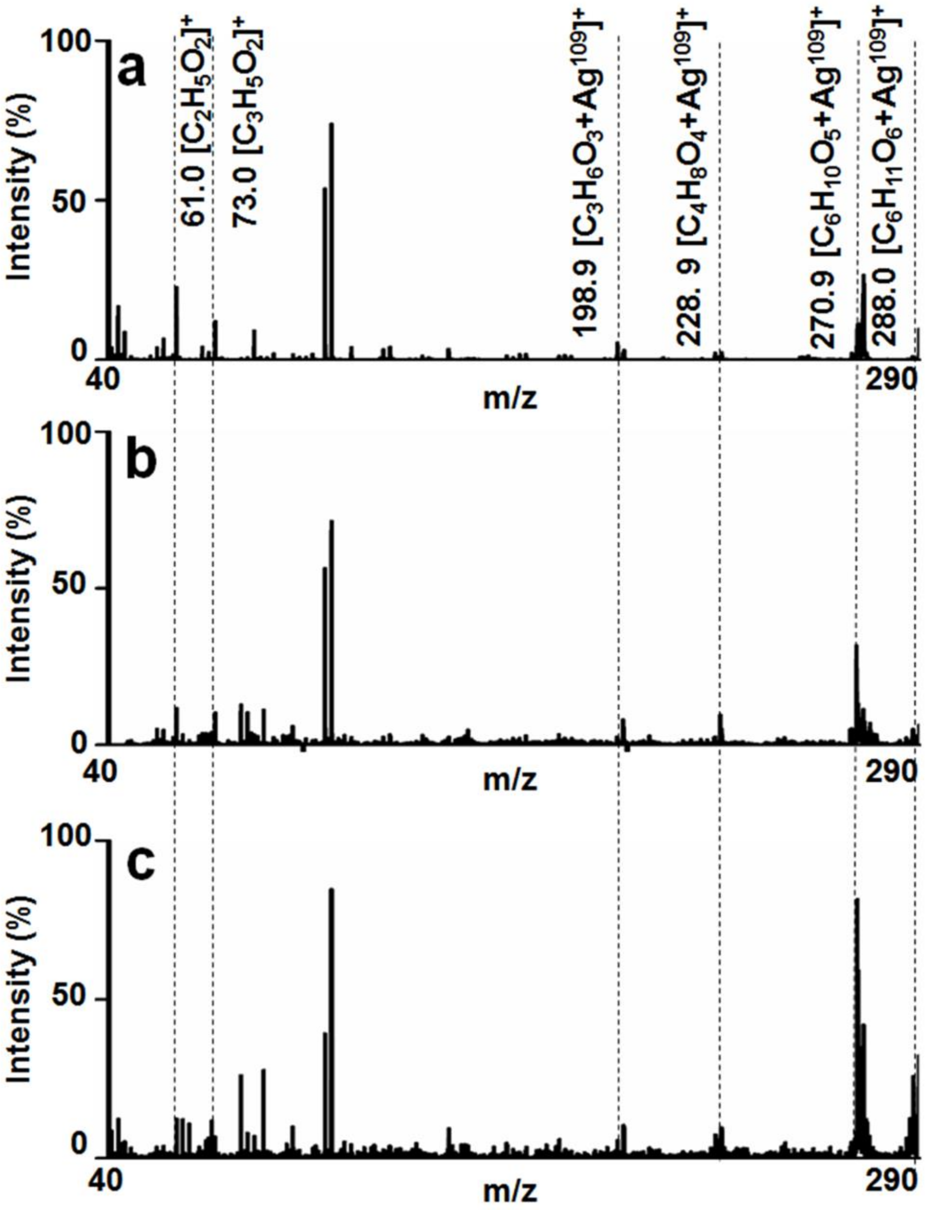


**Supplementary Fig. 17. Tandem mass spectra of silver adducted mannitol.** MS/MS of mannitol at m/z of 291.2 for $[M+^{109}Ag]^+$ in a) standard sample, b) CSF, and c) serum.

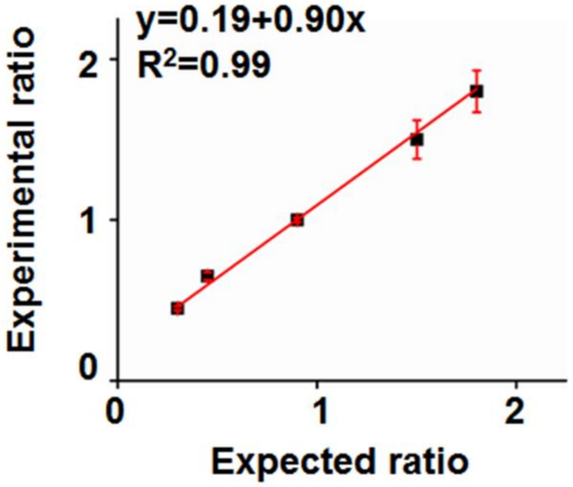


**Supplementary Fig. 18. The calibration curve obtained by plotting experimental ratio of analyte/isotope (A/I) as a function of expected ratio of A/I for glucose.** The isotope (IS) contained one $^{13}C$. 5 independent experiments were performed for each sample to calculate the standard deviation (s.d.) as error bars. Data were shown as the mean ±s.d. (n=5).

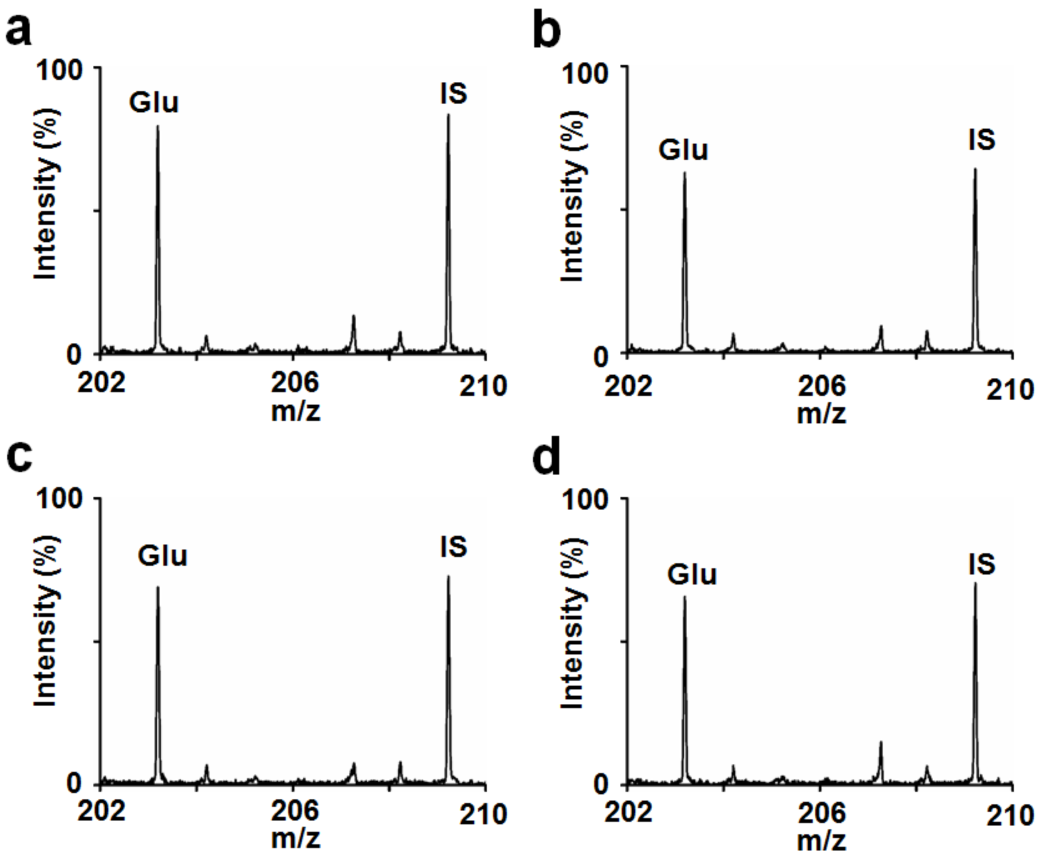


**Supplementary Fig. 19. Quantitation of glucose using isotope.** Typical mass spectra of glucose and its isotope for quantification by the other 4 independent experiments (in addition to Figure 3d) in parallel (A/I, 1/1).

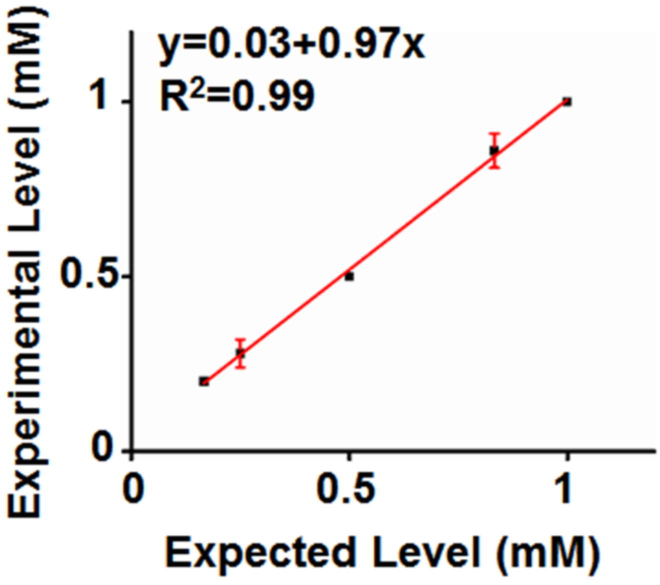


**Supplementary Fig. 20. The calibration curve by the biochemical method.** 5 independent experiments were performed for each sample to calculate the standard deviation (s.d.) as error bars. Data were shown as the mean ±s.d. (n=5).

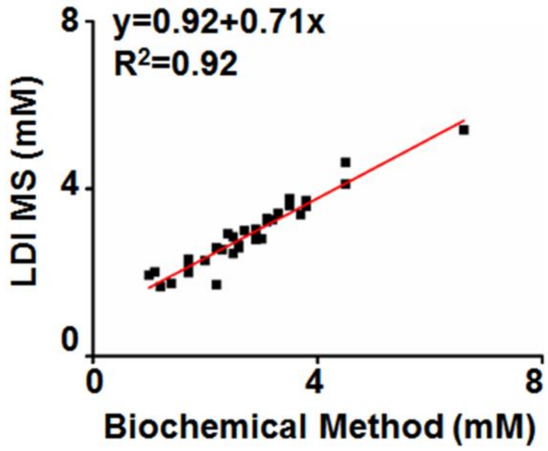


**Supplementary Fig. 21. Linear correlation**. Quantification results from the biochemical method and LDI MS, affording $R^2$ of 0.92.

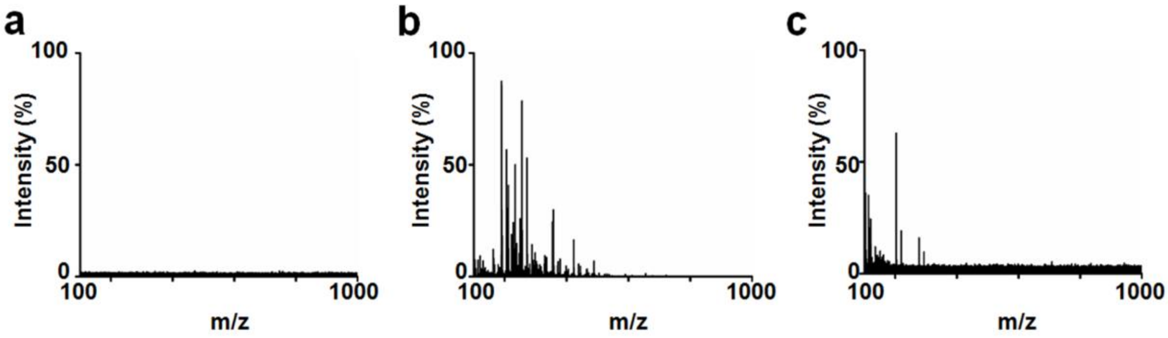


**Supplementary Fig. 22. Serum detection using other matrices.** Mass spectra of 500 nL of native serum using a) no matrix; b) CHCA; c) gold nanoparticles in the positive ion mode.

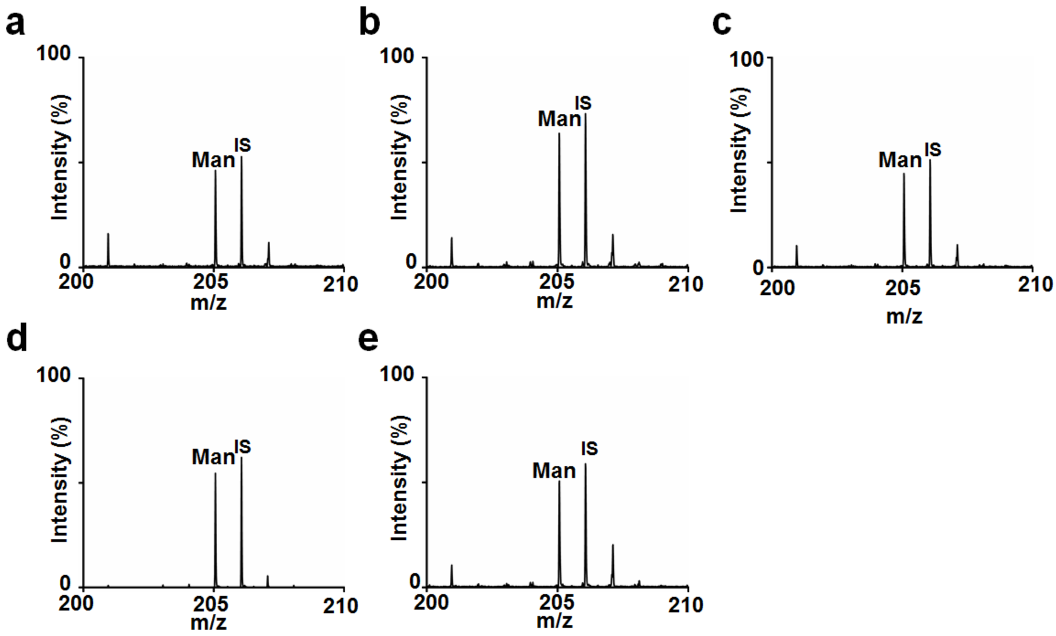


**Supplementary Fig. 23. Quantitation of mannitol using isotope.** Mass spectra of mannitol and its isotope for quantification by 5 independent experiments in parallel. The isotope (IS) contained one $^{13}C$.

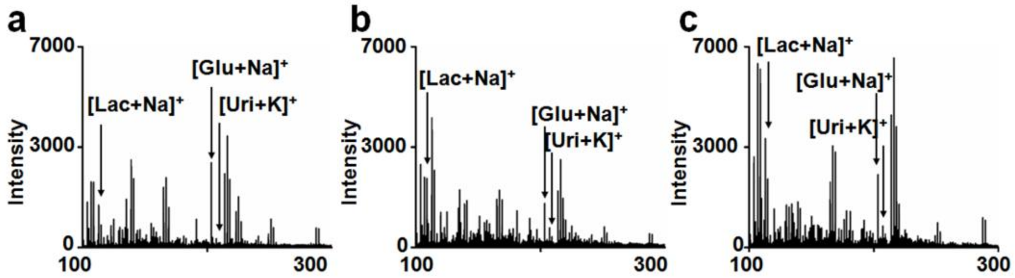


**Supplementary Fig. 24. Stability of silver nanoshells for serum detection.** Mass spectra of native serum using a) newly prepared $SiO_2$@Ag-3, b) $SiO_2$@Ag-3 stored for 2 months, and c) $SiO_2$@Ag-3 stored for 5 months. For clarity, only some of the signals were labeled.

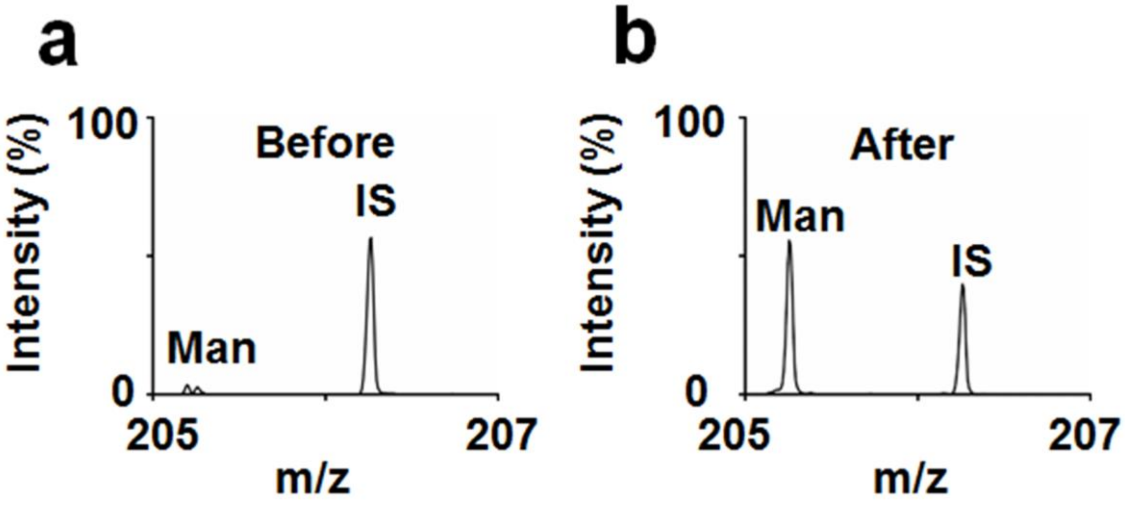


**Supplementary Fig. 25. Typical mass spectra of mannitol and its isotope in serum from a patient undergoing mannitol treatment.** The figures showed mannitol levels a) before injection and b) 30 min after injection.

## Supplementary Table 1 Structural parameters of the particles

| Samples | Average Size (nm)[a] | PDI[a] | Zeta Potential (mV) | Surface Area ($m^2$ $g^{-1}$) [b] |
|---|---|---|---|---|
| $SiO_2$ | 181.8±12.53 | 0.203±0.112 | -40.7±0.681 | 18.58±0.095 |
| $SiO_2$@Ag-1 | 185.8±0.611 | 0.105±0.064 | -37.4±1.760 | 24.20±0.069 |
| $SiO_2$@Ag-2 | 194.6±2.007 | 0.190±0.008 | -25.9±1.650 | 21.17±0.168 |
| $SiO_2$@Ag-3 | 210.8±9.717 | 0.202±0.020 | -22.2±1.130 | 20.72±0.115 |
| $SiO_2$@Ag-4 | 289.7±74.19 | 0.313±0.044 | -26.7±0.520 | 21.15±0.110 |

[a] The measurements of particle size and PDI were according to the DLS experiments.

[b] The surface areas were obtained in nitrogen adsorption analysis.

**Supplementary Table 2 Signal intensities of silver adducted signals for $SiO_2$@Ag-1/2/3/4**

| Small molecules | | $SiO_2$@Ag-1 | $SiO_2$@Ag-2 | $SiO_2$@Ag-3 | $SiO_2$@Ag-4 |
|---|---|---|---|---|---|
| Mannitol | m/z $[M+^{107}Ag]^+$ | 2077.0±337.36 | 2618.0±682.12 | 5393.7±279.39 | 4131.3±67.61 |
| | m/z $[M+^{109}Ag]^+$ | 1952.7±296.84 | 2535.3±685.89 | 5146.7±294.88 | 3933.7±58.02 |
| Glucose | m/z $[M+^{107}Ag]^+$ | 587.3±58.91 | 2407.0±208.23 | 5240.7±777.72 | 1629.3±385.92 |
| | m/z $[M+^{109}Ag]^+$ | 535.0±49.93 | 2219.7±174.17 | 4899.3±762.50 | 1516.0±370.01 |
| Methionine | m/z $[M+^{107}Ag]^+$ | 190.7±19.14 | 1011.3±227.99 | 2035.3±300.48 | 783.7±411.72 |
| | m/z $[M+^{109}Ag]^+$ | 182.7±13.07 | 991.7±221.93 | 1994.7±288.59 | 776.3±408.46 |

**Supplementary Table 3 m/z values of characteristic molecular peaks of typical metabolites by $SiO_2$@Ag assisted LDI MS**

| Small molecules | m/z $[M+Na]^+$ | m/z $[M+K]^+$ | m/z $[M+^{107}Ag]^+$ | m/z $[M+^{109}Ag]^+$ |
|---|---|---|---|---|
| Methionine | 172.21 | 188.20 | 256.20 | 258.20 |
| Glucose | 203.26 | 219.22 | 287.23 | 289.23 |
| Mannitol | 205.28 | 221.27 | 289.26 | 291.26 |

**Supplementary Table 4 m/z values of characteristic molecular peaks of typical metabolites from CSF by $SiO_2$@Ag assisted LDI MS**

| Small molecules | m/z $[M+Na]^+$ | m/z $[M+K]^+$ | m/z $[M+^{107}Ag]^+$ | m/z $[M+^{109}Ag]^+$ |
|---|---|---|---|---|
| Glucose | 203.26 | 219.22 | 287.23 | 289.23 |
| Mannitol | 205.28 | 221.27 | 289.23 | 291.23 |
| Uric acid | 197.07 | 207.21 | 275.36 | 277.33 |
| Tryptophan | 227.04 | 243.30 | 311.01 | 313.01 |

**Supplementary Table 5. Glucose levels from 38 CSF samples obtained by biochemical analyzer and particle assisted LDI MS**

| Sample number | Glucose detected by biochemical analyzer (mM) | Glucose detected by particle assisted LDI MS (mM) |
|---|---|---|
| 1 | 2.5 | 2.5 |
| 2 | 2.4 | 2.9 |
| 3 | 3 | 2.8 |
| 4 | 3.3 | 3.4 |
| 5 | 3.8 | 3.7 |
| 6 | 3.1 | 3.2 |
| 7 | 1.7 | 2.2 |
| 8 | 3.7 | 3.4 |
| 9 | 3.8 | 3.6 |
| 10 | 1.7 | 2.3 |
| 11 | 3.2 | 3.3 |
| 12 | 6.6 | 5.4 |
| 13 | 4.5 | 4.6 |
| 14 | 1.1 | 2.0 |
| 15 | 1 | 1.9 |
| 16 | 1.2 | 1.7 |
| 17 | 1.4 | 1.7 |
| 18 | 2.6 | 2.7 |
| 19 | 2.9 | 3.0 |
| 20 | 2.9 | 2.8 |
| 21 | 1.7 | 2.0 |
| 22 | 2.9 | 2.8 |
| 23 | 2.9 | 2.8 |
| 24 | 3.1 | 3.2 |
| 25 | 3.5 | 3.8 |
| 26 | 2.2 | 2.6 |
| 27 | 2.6 | 2.6 |
| 28 | 2.5 | 2.8 |
| 29 | 2.2 | 1.7 |
| 30 | 2.9 | 2.9 |
| 31 | 3.5 | 3.6 |
| 32 | 2.9 | 2.9 |
| 33 | 2 | 2.3 |
| 34 | 4.5 | 4.1 |
| 35 | 2.9 | 3.0 |
| 36 | 2.3 | 2.5 |
| 37 | 3.1 | 3.3 |
| 38 | 2.7 | 3.0 |

**Supplementary Table 6 m/z values of characteristic molecular peaks of typical metabolites from serum by $SiO_2$@Ag assisted LDI MS**

| Small molecules | m/z $[M+Na]^+$ | m/z $[M+K]^+$ | m/z $[M+^{107}Ag]^+$ | m/z $[M+^{109}Ag]^+$ |
|---|---|---|---|---|
| Glucose | 203.26 | 219.22 | 287.23 | 289.23 |
| Mannitol | 205.28 | 221.27 | 289.23 | 291.23 |
| Lactic acid | 113.03 | 129.10 | 197.07 | 199.07 |

**Supplementary Table 7. Mannitol level in CSF and serum samples from 3 patients before and 30 min after intravenous injection obtained by silver nanoshells assisted LDI MS**

| Patient number | Mannitol in CSF (mM) | | Mannitol in serum (mM) | |
|---|---|---|---|---|
| | Before | After | Before | After |
| 1 | 0.086±0.014 | 0.109±0.015 | 0.078±0.018 | 4.239±0.067 |
| 2 | 0.011±0.0001 | 0.026±0.002 | 0.100±0.018 | 5.198±0.260 |
| 3 | 0.019±0.002 | 0.025±0.004 | 0.087±0.043 | 4.022±0.151 |
